# Erythrocyte and Porcine Intestinal Glycosphingolipids Recognized by F4 Fimbriae of Enterotoxigenic *Escherichia coli*

**DOI:** 10.1371/journal.pone.0023309

**Published:** 2011-09-16

**Authors:** Annelies Coddens, Erik Valis, John Benktander, Jonas Ångström, Michael E. Breimer, Eric Cox, Susann Teneberg

**Affiliations:** 1 Laboratory of Veterinary Immunology, Faculty of Veterinary Medicine, Ghent University, Merelbeke, Belgium; 2 Institute of Biomedicine, Department of Medical Biochemistry and Cell Biology, University of Gothenburg, Göteborg, Sweden; 3 Department of Surgery, Sahlgrenska University Hospital, Göteborg, Sweden; Universität Münster, Germany

## Abstract

Enterotoxigenic F4-fimbriated *Escherichia coli* is associated with diarrheal disease in neonatal and postweaning pigs. The F4 fimbriae mediate attachment of the bacteria to the pig intestinal epithelium, enabling an efficient delivery of diarrhea-inducing enterotoxins to the target epithelial cells. There are three variants of F4 fimbriae designated F4ab, F4ac and F4ad, respectively, having different antigenic and adhesive properties. In the present study, the binding of isolated F4ab, F4ac and F4ad fimbriae, and F4ab/ac/ad-fimbriated *E. coli*, to glycosphingolipids from erythrocytes and from porcine small intestinal epithelium was examined, in order to get a comprehensive view of the F4-binding glycosphingolipids involved in F4-mediated hemagglutination and adhesion to the epithelial cells of porcine intestine. Specific interactions between the F4ab, F4ac and F4ad fimbriae and both acid and non-acid glycosphingolipids were obtained, and after isolation of binding-active glycosphingolipids and characterization by mass spectrometry and proton NMR, distinct carbohydrate binding patterns were defined for each fimbrial subtype. Two novel glycosphingolipids were isolated from chicken erythrocytes, and characterized as GalNAcα3GalNAcß3Galß4Glcß1Cer and GalNAcα3GalNAcß3Galß4GlcNAcß3Galß4Glcß1Cer. These two compounds, and lactosylceramide (Galß4Glcß1Cer) with phytosphingosine and hydroxy fatty acid, were recognized by all three variants of F4 fimbriae. No binding of the F4ad fimbriae or F4ad-fimbriated *E. coli* to the porcine intestinal glycosphingolipids occurred. However, for F4ab and F4ac two distinct binding patterns were observed. The F4ac fimbriae and the F4ac-expressing *E. coli* selectively bound to galactosylceramide (Galß1Cer) with sphingosine and hydroxy 24:0 fatty acid, while the porcine intestinal glycosphingolipids recognized by F4ab fimbriae and the F4ab-fimbriated bacteria were characterized as galactosylceramide, sulfatide (SO_3_-3Galß1Cer), sulf-lactosylceramide (SO_3_-3Galß4Glcß1Cer), and globotriaosylceramide (Galα4Galß4Glcß1Cer) with phytosphingosine and hydroxy 24:0 fatty acid. Finally, the F4ad fimbriae and the F4ad-fimbriated *E. coli*, but not the F4ab or F4ac subtypes, bound to reference gangliotriaosylceramide (GalNAcß4Galß4Glcß1Cer), gangliotetraosylceramide (Galß3GalNAcß4Galß4Glcß1Cer), isoglobotriaosylceramide (Galα3Galß4Glcß1Cer), and neolactotetraosylceramide (Galß4GlcNAcß3Galß4Glcß1Cer).

## Introduction

Adhesion of microbes and microbial toxins to their target tissue by binding to cell surface carbohydrates is nowadays textbook knowledge, as *e.g.* the binding of influenza virus to sialic acid-containing glycoconjugates, cholera toxin to the GM1 ganglioside, and uropathogenic P-fimbriated *Escherichia coli* to Galα4Gal-containing glycosphingolipids. However, for many microbial adhesins the target cell receptors have not yet been identified.

Enterotoxigenic *E. coli* (ETEC) infections are a major cause of diarrhea in young pigs. Infecting bacteria adhere to and colonize the intestinal epithelium and cause diarrhea primarily by the production of heat-labile and/or heat-stable enterotoxin (LT and ST respectively). Adherence is mediated by fimbrial structures, and porcine ETEC primarily express five types of fimbriae designated F4 (K88), F5 (K99), F6 (987P), F41 and F18. F4 fimbriae are the most prevalent fimbrial structures expressed by porcine ETEC causing diarrhea and mortality in newborn, suckling and newly weaned piglets [1]. The F4 fimbriae are composed of a large number of the major subunit FaeG, with a small number of minor subunits interspersed throughout the structure [2]. F4 fimbriae are expressed by the *fae*-operon, coding for the regulatory proteins FaeA and FaeB [3], the fimbrial tip protein FaeC [4], the usher FaeD [5], the chaperone FaeE [6], the minor fimbrial shaft subunits FaeF and FaeH [7], the adhesive major subunit FaeG [8], and the minor subunits FaeI and FaeJ with undefined roles.

There are three antigenically distinct variants of F4 fimbriae designated F4ab, F4ac and F4ad, respectively, distinguished by amino acid substitutions in the FaeG subunit [2], [9], [10]. Studies on the interactions of the variants of F4 fimbriae with erythrocytes, intestinal mucus and intestinal epithelial cells have demonstrated that the F4ab, F4ac and F4ad fimbriae have different, but related, carbohydrate binding specificities (reviewed in [2]), and several receptor candidates for each variant have been suggested. Studies of the hemagglutinating properties of F4ab-, F4ac- and F4ad-expressing *E. coli*, using a panel of erythrocytes from different species, showed that rabbit and guinea pig erythrocytes were agglutinated in a mannose-resistant manner by all three serotypes [8]. Pig and rat erythrocytes were agglutinated by F4ab- and F4ad-fimbriated *E. coli*, while chicken erythrocytes were selectively agglutinated by F4ab-fimbriated bacteria.

Previous studies of the carbohydrate recognition of F4ab-(K88ab-)fimbriated *E. coli* using reference glycosphingolipids, demonstrated a binding to galactosylceramide (Galß1Cer) with hydroxy ceramide, lactosylceramide (Galß4Glcß1Cer), gangliotriaosylceramide (GalNAcß4Galß4Glcß1Cer), and gangliotetraosylceramide (Galß3GalNAcß4Galß4Glcß1Cer), leading to the conclusion that a Galß1 was necessary for binding to occur [11]. Another glycosphingolipid binding study demonstrated binding of F4ab-, F4ac- and F4ad-fimbriae to lactotriaosylceramide (GlcNAcß3Galß4Glcß1Cer), neolactotetraosylceramide (Galß4GlcNAcß3Galß4Glcß1Cer), and neolactohexaosylceramide (Galß4GlcNAcß3Galß4GlcNAcß3Galß4Glcß1Cer), globotriaosylceramide (Galα4Galß4Glcß1Cer), gangliotri- and gangliotetraosylceramide [12]. By exoglycosidase digestion of porcine serum transferrin it was also demonstrated that the F4ab adhesin recognizes GlcNAc residues in the core chain of N-linked glycans. Taken together, these results lead to the conclusion that the minimal binding epitope for all three variants of the F4-fimbriae is a ß-linked HexNAc residue, and that an increased binding is obtained by substitution of the HexNAc with a terminal Galß. In addition, a porcine intestinal glycosphingolipid recognized by F4ad, but not by F4ab or F4ac, has been identified as neolactotetraosylceramide [13].

In order to get a comprehensive view of the F4-binding glycosphingolipids involved in F4-mediated hemagglutination and adhesion to the epithelial cells of porcine intestine, the binding of isolated F4 fimbriae, and F4-fimbriated *E. coli*, to glycosphingolipids from erythrocytes (human, chicken, guinea pig, rabbit and porcine) and from porcine small intestinal epithelium was examined in the present study. Specific interactions between the F4ab, F4ac and F4ad fimbriae and both acid and non-acid glycosphingolipids were obtained, and after isolation of binding-active glycosphingolipids and characterization by mass spectrometry and proton NMR, distinct binding patterns were defined for each fimbrial subtype.

## Results

### Characterization of native and mutant F4 fimbriae

Wild type and deletion mutant F4 fimbriae were isolated for glycosphingolipid binding experiments. When analyzed by SDS-PAGE the isolated F4 fimbriae variants all migrated as single bands, which represent the major subunit FaeG, and the apparent molecular weight of the proteins was in agreement with the predicted molecular masses (Figure S1) [2].

### Binding of F4 fimbriae and F4-fimbriated *E. coli* to erythrocyte glycosphingolipids

**Figure 1:**
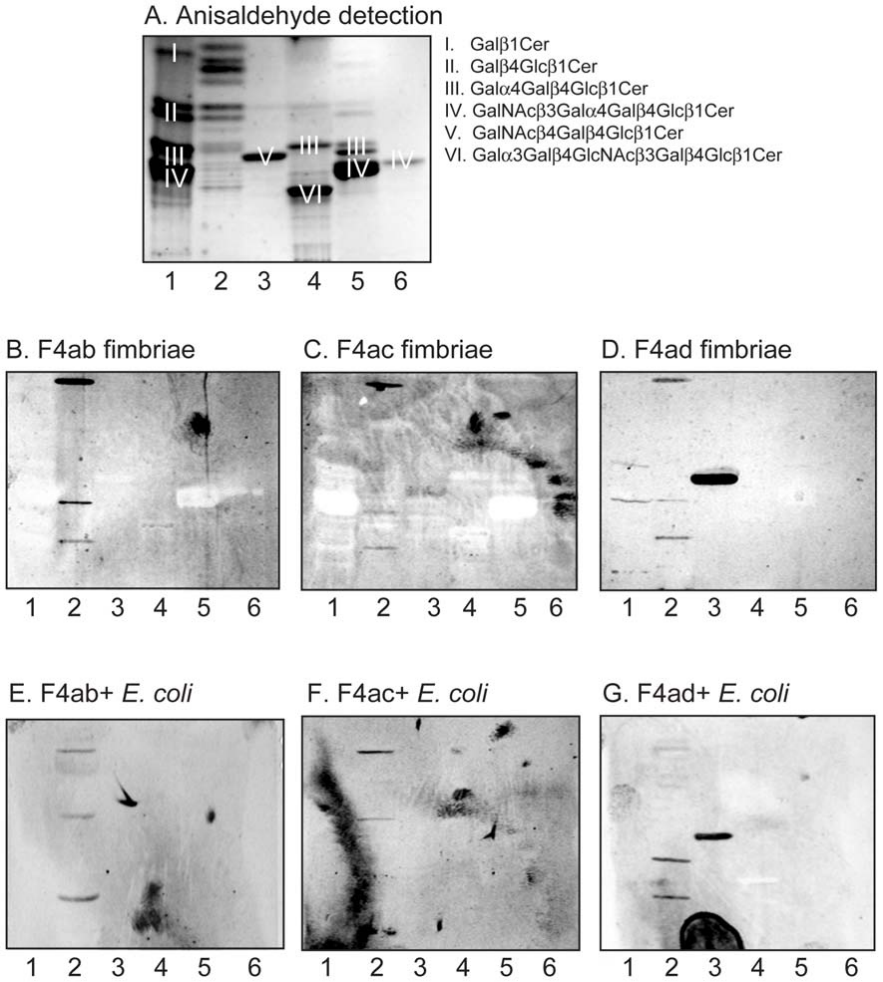
Binding of F4 fimbriae and F4-fimbriated *Escherichia coli* to erythrocyte non-acid glycosphingolipid mixtures. Chemical detection by anisaldehyde (A), and autoradiograms obtained by binding of ^125^I-labeled F4ab fimbriae (B), F4ac fimbriae (C), F4ad fimbriae (D), and ^35^S-labeled F4ab-expressing *E. coli* (E), F4ac-expressing *E. coli* (F), and F4ad-expressing *E. coli* (G). The lanes were: Lane 1, non-acid glycosphingolipids of human erythrocytes blood group AB, 80 µg; Lane 2, non-acid glycosphingolipids of chicken erythrocytes, 40 µg; Lane 3, non-acid glycosphingolipids of guinea pig erythrocytes, 40 µg; Lane 4, non-acid glycosphingolipids of rabbit erythrocytes, 40 µg; Lane 5, non-acid glycosphingolipids of porcine erythrocytes, 40 µg; Lane 6, reference globotetraosylceramide (GalNAcß3Galα4Galß4Glcß1Cer) of human erythrocytes, 4 µg. The major compounds visualized with anisaldehyde in (A) are marked with Roman numbers, and the corresponding glycosphingolipid structures are given to the right of the chromatogram.

The initial F4 binding studies were done using mixtures of total acid and non-acid glycosphingolipids isolated from human, chicken, guinea pig, rabbit and pig erythrocytes. No binding to the acid glycosphingolipid fractions was obtained (data not shown). However, all three fimbriae, and the three corresponding variants of F4-fimbriated bacteria, selectively bound to three compounds in the non-acid glycosphingolipid fraction of chicken erythrocytes (Fig. 1, lane 2). The binding-active compounds migrated in the mono-, tetra- and hexaglycosylceramide regions, respectively. In addition, the F4ad fimbriae and the F4ad-fimbriated *E.coli* distinctly bound to the major compound of guinea pig erythrocytes, migrating in the triglycosylceramide region (Fig. 1D and G, respectively, lane 3). This compound was not recognized by the F4ab or F4ac fimbriae or the bacteria expressing F4ab or F4ac fimbriae. The major glycosphingolipid of guinea pig erythrocytes is gangliotriaosylceramide (GalNAcß4Galß4Glcß1Cer) [14], and gangliotriaosylceramide isolated from this source was recognized by F4ad fimbriae and F4ad-expressing *E. coli* (see below).

### Isolation of the F4 fimbriae binding slow-migrating glycosphingolipids of chicken erythrocytes

**Figure 2:**
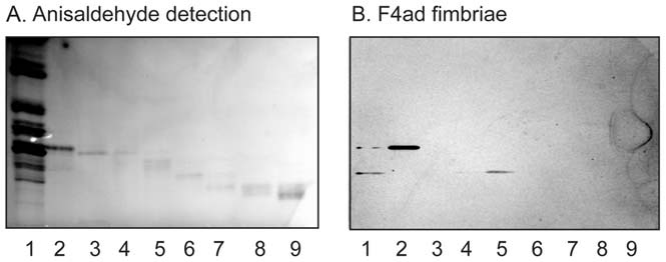
Binding of F4ad fimbriae to slow-migrating non-acid glycosphingolipid fractions isolated from chicken erythrocytes. Chemical detection by anisaldehyde (A), and autoradiograms obtained by binding of ^125^I-labeled F4ad fimbriae (B). The lanes were: Lane 1, non-acid glycosphingolipids of chicken erythrocytes, 40 µg; Lanes 2–9, glycosphingolipid fractions isolated from chicken erythrocytes, 0.5–2 µg/lane.

Previously characterized glycosphingolipids of chicken erythrocytes are galactosylceramide, lactosylceramide, and the Forssman pentaglycosylceramide (GalNAcα3GalNAcß3Galα4Galß4Glcß1Cer) [15]. Here we focused on the F4 binding glycosphingolipids of chicken erythrocytes migrating in the tetra- and hexaglycosylceramide regions. These F4-binding glycosphingolipids were isolated by chromatography on an Iatrobeads column, and the fractions obtained were tested for F4ab and F4ad binding activity using ^125^I-labeled fimbriae (exemplified for F4ad in Fig. 2B). After pooling of binding-active fractions, 0.5 mg of a fraction containing the binding-active compound migrating in the tetraglycosylceramide region (designated fraction C:tetra-I; Fig. 2, lane 2), and less than 100 µg of the binding-active hexaglycosylceramide (designated fraction C:hexa; Fig. 2, lane 5), were obtained. Proton NMR showed that fraction C:tetra-I was a mixture of four glycosphingolipids. This fraction was therefore further separated on an Iatrobeads column and, after pooling of the F4-binding fractions, 0.2 mg was obtained (designated fraction C:tetra-II).

### ESI/MS of fraction C:tetra-II

ESI/MS of the native fraction C:tetra-II gave a series of pseudomolecular ions [M-H^+^]^−^ at *m/z* 1282–1394, indicating a tetraglycosylceramide with two HexNAc and two Hex, and with sphingosine and hydroxy 16:0-24:0 fatty acids (data not shown). MS^2^ of the predominant [M-H^+^]^−^ ion at *m/z* 1282 gave a series of Y and Z ions identifying a glycosphingolipid with HexNAc-HexNAc-Hex-Hex sequence and with sphingosine and hydroxy 16:0 fatty acid (Figure S2).

### Capillary-LC/MS and MS/MS

**Figure 3:**
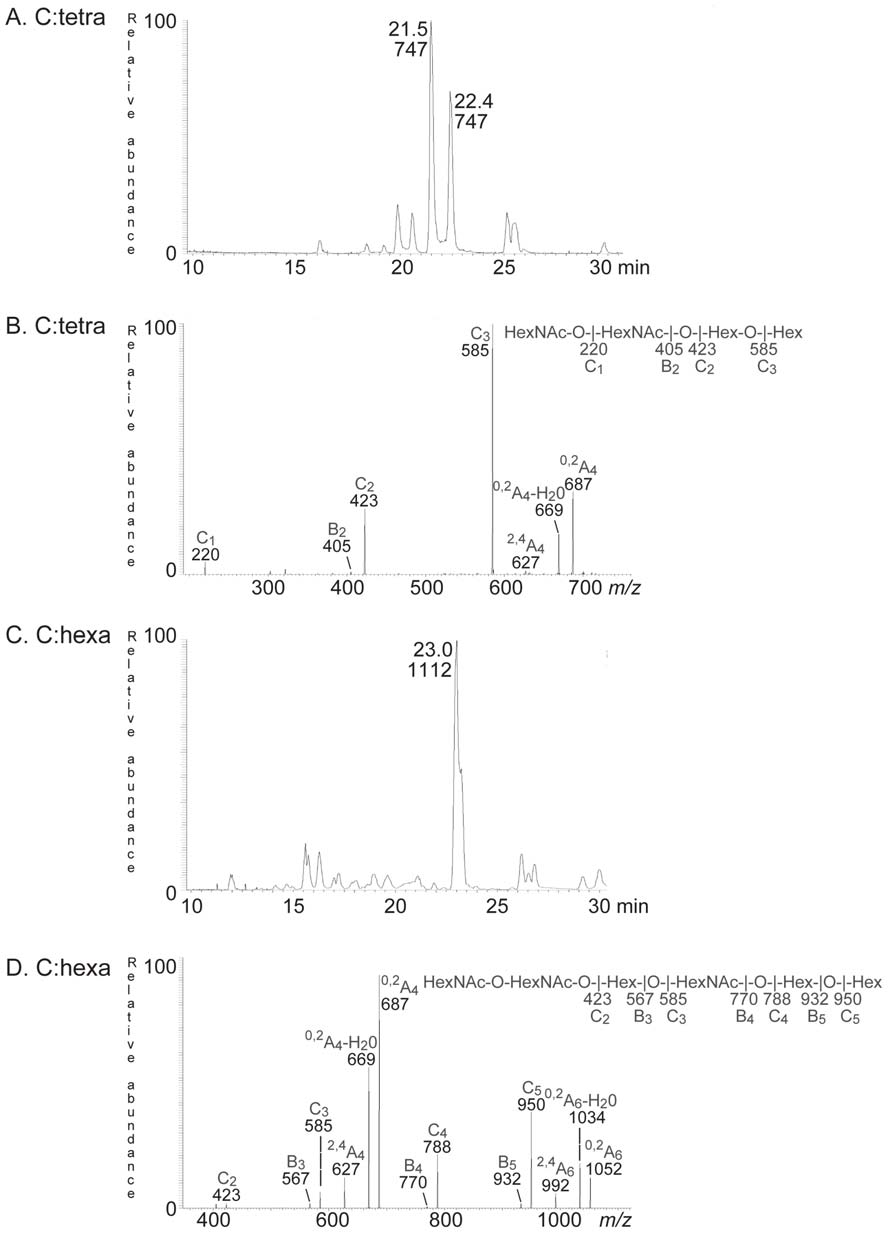
Characterization of the F4 fimbriae binding tetra- and hexaglycosylceramide of chicken erythrocytes. (A) Base peak chromatogram from LC-ESI/MS of the saccharide obtained by digestion with *Rhodococcus* endoglycoceramidase II of the F4-binding glycosphingolipid fraction C:tetra-II from chicken erythrocytes. (B) MS^2^ spectrum of the ion at *m/z* 747 (retention time 21.4 min). (C) Base peak chromatogram from LC-ESI/MS of the saccharide obtained by digestion with *Rhodococcus* endoglycoceramidase II of the F4-binding glycosphingolipid fraction C:hexa from chicken erythrocytes. (D) MS^2^ spectrum of the ion at *m/z* 1112 (retention time 23.0 min).

The oligosaccharides obtained from fraction C:tetra-II by hydrolysis with *Rhodococcus* endoglycoceramidase II, were analyzed by LC-ESI/MS using a graphitized carbon column [16]. The major saccharide of this fraction was detected as a [M-H]^−^ ion at *m/z* 747, eluting at 21.5–22.4 min (Fig. 3A). MS^2^ of the [M-H]^−^ ion at *m/z* 747 resulted in a series of prominent C-type fragment ions (C_1_ at *m/z* 220, C_2_ at *m/z* 423, and C_3_ at *m/z* 585) identifying a tetrasaccharide with HexNAc-HexNAc-Hex-Hex sequence (Fig. 3B). The ^0,2^A_4_ ion at *m/z* 687 and the ^0,2^A_4_-H_2_O ion at *m/z* 669 were obtained by cross-ring cleavages of the 4-substituted Glc of the internal lactose (Galß4Glc) part. However, no other cross-ring cleavage ions were observed, suggesting that the two terminal HexNAcs were 3-linked [16].

The major saccharide derived from fraction C:hexa gave a [M-H]^−^ ion at *m/z* 1112, eluting at 23.0–23.2 min (Fig. 3C). The MS^2^ spectrum had a series of C-type fragment ions (C_2_ at *m/z* 423, C_3_ at *m/z* 585, C_4_ at *m/z* 787 and C_5_ at *m/z* 950) demonstrating a hexasaccharide with HexNAc-HexNAc-Hex-HexNAc-Hex-Hex sequence (Fig. 3D). The prominent cross-ring ^0,2^A_4_ fragment ion at *m/z* 687, and the accompanying ^0,2^A_4_-H_2_O fragment ion at *m/z* 669, indicated a 4-substitution of the internal HexNAc, *i.e.* a type 2 core [16]. Again, the absence of cross-ring cleavage ions suggested that the two terminal HexNAcs were 3-linked.

### Proton NMR spectroscopy of fractions C:tetra-I, C:tetra-II and C:hexa

**Figure 4:**
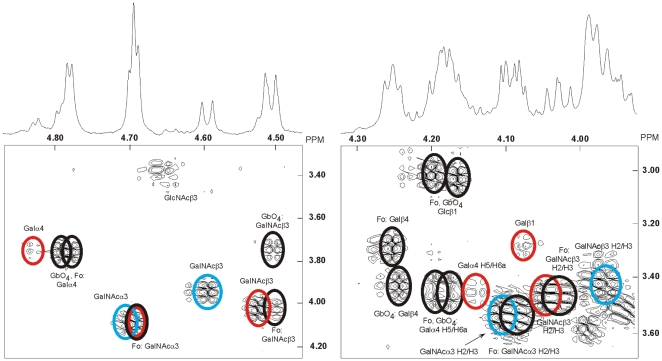
Proton NMR of the F4-binding glycosphingolipid (fraction C:tetra-I) from chicken erythrocytes. Anomeric regions of the 600 MHz proton NMR of the F4-binding glycosphingolipid (fraction C:tetra-I) from chicken erythrocytes (30°C). The sample was dissolved in dimethyl sulfoxide-D_2_O (98∶2, by volume) after deuterium exchange. Below each section the corresponding DQF-COSY spectrum showing mainly the H1/H2 connectivities are displayed. Connectivities stemming from the same structure are color-coded by superimposed ellipses. Thus, the Forssman pentaglycosylceramide (Fo; GalNAcα3GalNAcß3Galα4Galß4Glcß1Cer) and globoside (GbO_4_; GalNAcß3Galα4Galß4Glcß1Cer) are black, whereas the two novel four-sugar compounds B and C are colored blue and red, respectively. Furthermore, only connectivities other than H1/H2 ones are denoted as such.

**Figure 5:**
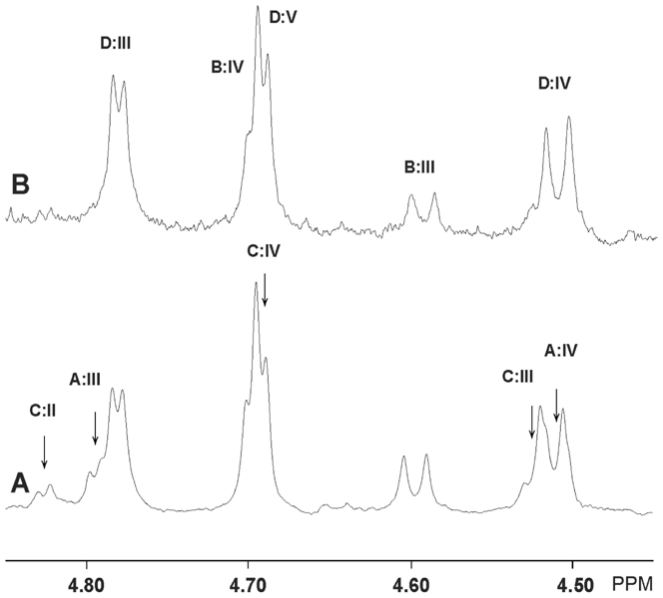
Proton NMR of fraction C:tetra-I and fraction C-tetra-II from chicken erythrocytes. Low-field part of the anomeric regions of the 600 MHz spectra of fraction C:tetra-I (A) and fraction C-tetra-II (B). The arrows in (A) indicate which resonances that have disappeared in (B) and these are labeled according to the scheme in Table 1. Likewise, the remaining resonances in (B) are also labeled as in Table 1.

The anomeric region of the 600 MHz spectrum of fraction C:tetra-I is shown as two partial spectra on top of the corresponding COSY sections in Fig. 4, and in Fig. 5 the low field portion of the anomeric region (A; C:tetra-I) is compared to the corresponding section of the same fraction having been further purified (B; C:tetra-II). From Figs. 4 and 5, and the spectrum of the following fraction C:penta (not shown), which almost exclusively turned out to contain the Forssman pentaglycosylceramide (GalNAcα3GalNAcß3Galα4Galß4Glcß1Cer) [17], it is concluded that the spectrum in Fig. 5B represents two different species, one of which is the Forssman pentaglycosylceramide (labeled D), and a second one representing a novel four-sugar compound (labeled B) to be characterized below. It can be further concluded that the C:tetra-I fraction shown in Figs. 4 and 5A contains two additional minor species besides compounds B and D (labeled A and C, respectively). Compound A is easily identified from the NMR literature [17], mass spectrometry data, and the present COSY data, as globoside (GalNAcß3Galα4Galß4Glcß1Cer). The terminal GalNAcß3 is thus clearly separated from internal ones by the H1/H2 connectivity seen at 4.509/3.75 ppm (see Table 1), as opposed to the corresponding values seen *e.g.* for the GalNAcß3 of the Forssman pentaglycosylceramide (4.511/4.03 ppm) [17]. Furthermore, the anomeric resonances of the Galα4 residues stemming from globoside and the Forssman pentaglycosylceramide are seen as expected at 4.796 ppm and 4.781 ppm, respectively, whereas the terminal GalNAcα3 of the Forssman pentaglycosylceramide is readily found at 4.692 ppm, overlapping with another GalNAcα3 anomeric resonance from the novel B compound. The H1/H2 connectivities arising from the Galß4Glcß1 segments of these two compounds are also clearly revealed as seen in the right panel of Fig. 4.

**Table 1:** Summary of glycosphingolipid structures identified by NMR in the 4–6 sugar region of fractions C:tetra-I, C:tetra-II, and C:hexa in chicken erythrocytes (the identity of the anomeric and other ring proton resonances are given in the NMR spectra shown in Figs. 4 and 5).

Structure	Trivial name	VI	V	IV	III	II*	I*	
**A**	Globoside			GalNAcß3	Galα4	Galß4	Glcß1	Cer
H1				4.509	4.796	4.244	4.165	
H2				3.75	3.76	3.43	3.03	
H3				3.48				
**B**				GalNAcα3	GalNAcß3	Galß4	Glcß1	Cer
H1				4.699	4.594	—	—	
H2				4.10	3.95			
H3				3.55	3.44			
**C**				GalNAcα3	GalNAcß3	Galα4	Galß1	Cer
H1				∼4.70	4.524	4.827	4.077	
H2					4.03	3.75	3.29	
H3					3.48			
**D**	Forssman pentaglycosylceramide		GalNAcα3	GalNAcß3	Galα4	Galß4	Glcß1	Cer
H1			4.692	4.511	4.781	4.254	4.195	
H2			4.10	4.03	3.76	3.29	3.02	
H3			3.55	3.48				
**E**	Extended x_2_	GalNAcα3	GalNAcß3	Galß4	GlcNAcß3	Galß4	Glcß1	Cer
H1		4.70	4.596	∼4.26	4.65	∼4.26	—	

*Chemical shift values are in some cases not given due to low intensities and severe resonance overlap.

From the mass spectrometry data discussed above it was concluded that the four-sugar compound has the sequence HexNAc-HexNAc-Hex-Hex with both HexNAc residues being 3-linked. Inspection of Fig. 5B shows that the novel compound contains one α- and one ß-HexNAc at ppm values (4.699 ppm and 4.594 ppm, respectively) consistent with their identity being a terminal GalNAcα3 and an internal GalNAcß3. The H1/H2 connectivity of the GalNAcß3 residue is only consistent with a penultimate position for this sugar residue (Table 1). Furthermore, a 4-linked GalNAc can be excluded since the preceding Galß4 residue would in this case be expected to display an H1/H2 connectivity around 4.21/3.23 ppm as in *e.g.* gangliotetraosylceramide (Galß3GalNAcß4Galß4Glcß1Cer) [18]. However, no such connectivity is observed in Fig. 4. The two remaining residues (Hex-Hex) are most likely represented by a Galß4Glcß1 segment where the anomeric resonances, however, are obscured by the corresponding resonances from globoside and the Forssman pentaglycosylceramide. The sequence of compound B is thus concluded to be GalNAcα3GalNAcß3Galß4Glcß1Cer.

Further inspection of Fig. 5A reveals three minor additional anomeric resonances yet to be assigned to specific residues: the one to lowest field is an α-signal at 4.827 ppm revealing an H2 signal at 3.75 ppm thus indicating a Galα4 residue [19]; the second one is a ß-signal at 4.645 ppm having the H2 resonance at 3.40 ppm, which identifies this sugar as a GlcNAcß3 residue which probably stems from a small percentage of neolactohexaosylceramide (the closeby intensity seen at 4.66/3.37 ppm in Fig. 4 is an artefact due to the residual HDO resonance); the third one is also a ß-signal seen partially overlapping the GalNAcß3 resonances stemming from globoside and the Forssman pentaglycosylceramide on the low-field side. This resonance is more clearly seen in the COSY spectrum and the H1/H2 connectivity at 4.524/4.03 ppm identifies also this sugar as an internal GalNAcß3 residue. The observation that the Galα4 H1 resonance is shifted almost 0.3 ppm to lower field is suggestive of an extended galabiaosylceramide based four-sugar structure. Galabiaosylceramide has previously been identified in chicken erythrocytes [15] and characterized by NMR [20]. In such a case the H1/H2 connectivity of Galß1 is expected around 4.08/4.29 ppm, which is exactly where a cross-peak of appropriate intensity is seen in Fig. 4. In the absence of a clearly distinguishable anomeric resonance that may conclusively identify the sugar in the fourth position, it may be inferred that a GalNAcα3 residue whose resonances completely overlap those of the corresponding residue in the Forssman antigen is the only viable assumption, thus suggesting that the final structure should be GalNAcα3GalNAcß3Galα4Galß1Cer (compound C). From a mass spectrometry point of view this sequence also explains why only one four-sugar compound could be detected.

NMR data for the C:hexa fraction was also obtained (not shown), but due to the low amount of material in this case, the spectral quality was rather poor. However, the signal to noise ratio was sufficient enough in order to clearly identify the Forssman pentaglycosylceramide, along with a second compound having three HexNAc residues whose identities are GalNAcα3 at 4.70 ppm, GlcNAcß3 at 4.65 ppm and GalNAcß3 at 4.596 ppm. The mass spectrometry of fraction C:hexa revealed the presence of the sequence HexNAc-HexNAc-Hex-HexNAc-Hex-Hex. The two residues closest to the ceramide are most likely Galß4Glcß1, which leaves the third Hex that most likely can be assigned to a second Galß4 residue. Taken together these data allows the structure to be identified as GalNAcα3GalNAcß3Galß4GlcNAcß3Galß4Glcß1Cer (compound E). This novel glycosphingolipid is thus the x_2_ glycosphingolipid elongated by a GalNAcα3 residue. The x_2_ glycosphingolipid has previously been characterized by NMR [21], and the chemical shifts are in accord with the ones found here (Table 1). It is noteworthy that the shifts of the two terminal HexNAc residues are identical to the ones found for compound B.

Thus, by mass spectrometry and proton NMR the F4-binding fraction C:tetra-II was characterized by the presence of the Forssman pentaglycosylceramide (GalNAcα3GalNAcß3Galα4Galß4Glcß1Cer) and GalNAcα3GalNAcß3Galß4Glcß1Cer, and the F4-binding fraction C:hexa by the presence of the Forssman pentaglycosylceramide and GalNAcα3GalNAcß3Galß4GlcNAcß3Galß4Glcß1Cer. GalNAcα3GalNAcß3Galß4Glcß1Cer, GalNAcα3GalNAcß3Galß4GlcNAcß3Galß4Glcß1Cer, and the GalNAcα3GalNAcß3Galα4Galß1Cer found in fraction C:tetra-I, are novel glycosphingolipid structures. Since no binding of the F4 fimbriae to reference Forssman pentaglycosylceramide was obtained (see below), GalNAcα3GalNAcß3Galß4Glcß1Cer and GalNAcα3GalNAcß3Galß4GlcNAcß3Galß4Glcß1Cer are the tetra- and hexaosylceramides recognized by the F4 fimbriae, and the GalNAcα3GalNAcß3Galß sequence is the minimum epitope required for binding to occur. Potentially, the minor GalNAcα3GalNAcß3Galα4Galß1Cer of fraction C:tetra:I could also be recognized by the F4 fimbriae. However, since the terminal trisaccharide of this tetraglycosylceramide is identical with the terminal trisaccharide of the non-binding Forssman pentaglycosylceramide, a binding of F4 fimbriae to GalNAcα3GalNAcß3Galα4Galß1Cer is less likely.

### Binding of F4 fimbriae and F4-fimbriated *E. coli* to glycosphingolipids of pig intestinal epithelium

**Figure 6:**
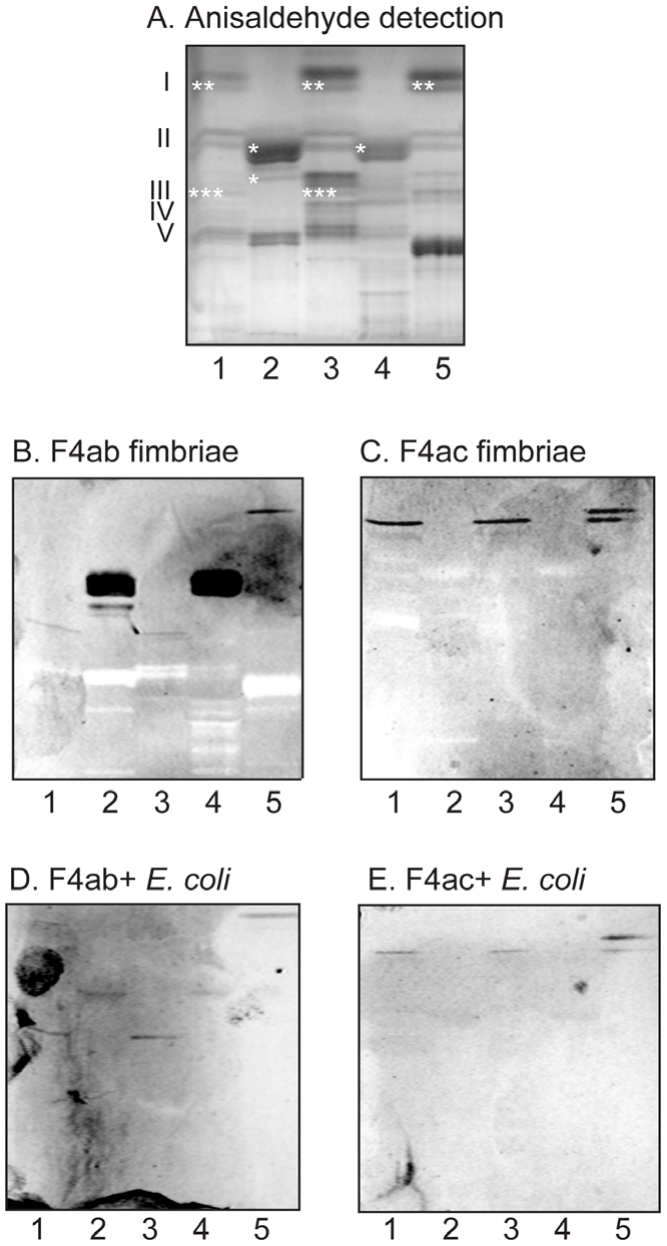
Binding of F4ab and F4ac fimbriae, and F4ab- and F4ac-fimbriated *Escherichia coli*, to mixtures of glycosphingolipids from porcine intestinal mucosa. Chemical detection by anisaldehyde (A), and autoradiograms obtained by binding of F4ab fimbriae (B), F4ac fimbriae (C), F4ab-expressing *E. coli* (D), and F4ac-expressing *E. coli* (E). The lanes were: Lane 1, non-acid glycosphingolipids of 3-day old piglet small intestinal mucosa, 40 µg; Lane 2, acid glycosphingolipids of 3-day old piglet small intestinal mucosa, 20 µg; Lane 3, non-acid glycosphingolipids of adult pig 1 small intestinal mucosa, 40 µg; Lane 4, acid glycosphingolipids of adult pig 1 small intestinal mucosa, 40 µg; Lane 5, non-acid glycosphingolipids of adult pig 2 small intestinal mucosa, 40 µg. The Roman numbers to the left of panel A indicate the approximate number of carbohydrate residues in the bands in the non-acid fractions (lanes 1, 3 and 5). The approximate migration level of the F4ab-/F4ac-binding compounds have been marked with *, **, and *** in panel A.

Thereafter, the binding of F4ab, F4ac and F4ad fimbriae and F4-fimbriated *E. coli* to mixtures of total acid and non-acid glycosphingolipids isolated from mucosal scrapings of newborn piglet and adult pig intestines was tested. No binding of the F4ad fimbriae or F4ad-expressing *E. coli* to the glycosphingolipids of pig intestinal epithelium occurred (not shown). In contrast, a specific binding of the F4ab fimbriae and the F4ab-fimbriated bacteria to some fast-migrating glycosphingolipids in the acid fractions was observed (Fig. 6B and C, lanes 2 and 4, marked with * in 6A). In the non-acid fractions, a compound migrating in the monoglycosylceramide region was recognized by both the F4ab and the F4ac fimbriae and the bacterial cells (Fig. 6, lanes 1, 2 and/or 5, marked with ** in 6A). In addition, the F4ab fimbriae and the F4ab-expressing *E. coli* recognized a compound migrating in the triglycosylceramide region (Fig. 6B and C, lanes 1 and 3, marked with *** in 6A).

### Characterization of the acid porcine intestinal glycosphingolipids recognized by F4ab fimbriae

In order to characterize the F4ab-binding acid glycosphingolipids of newborn piglet intestinal epithelium, this fraction was analyzed by TLC-FAB-MS (Figure S3). Thereby, the more slow-migrating F4ab-binding glycosphingolipid was tentatively identified as sulfated dihexosylceramide, while the fast-migrating F4ab-binding glycosphingolipid was tentatively identified as sulfated monohexosylceramide.

**Figure 7:**
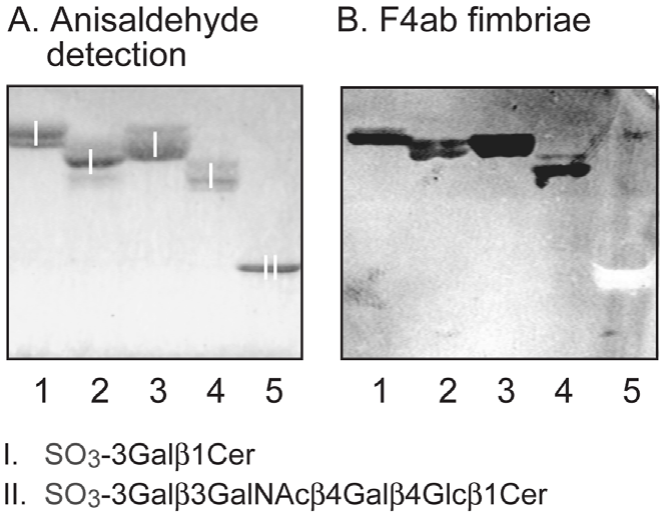
Binding of F4ab fimbriae to reference glycosphingolipids. Chemical detection by anisaldehyde (A), and autoradiograms obtained by binding of ^125^I-labeled F4ab fimbriae (B). The lanes were: Lane 1, sulfatide (SO_3_-3Galß1Cer) with d18:1-24:1 ceramide, 4 µg; Lane 2, sulfatide with d18:1-h16:0 ceramide, 4 µg; Lane 3, sulfatide with t18:0-h24:0 ceramide, 4 µg; Lane 4, sulf-lactosylceramide (SO_3_-3Galß4Glcß1Cer) with t18:0-h16:0 ceramide, 4 µg; Lane 5, reference sulf-gangliotetraosylceramide (SO_3_-3Galß3GalNAcß4Galß4Glcß1Cer), 4 µg. The glycosphingolipids visualized with anisaldehyde in (A) are marked with Roman numbers, and the corresponding glycosphingolipid structures are given below the chromatograms.

To confirm the identity of the sulfated glycosphingolipids recognized by the F4ab fimbriae, the binding of these fimbriae to a panel of reference sulfated glycosphingolipids was next evaluated (Fig. 7, and summarized in Table 2). Here the F4ab fimbriae recognized sulfatide (SO_3_-3Galß1Cer) with variant ceramide composition (Fig. 7, lanes 1–3), along with binding to sulf-lactosylceramide (SO_3_-3Galß4Glcß1Cer; lane 4), while no binding to sulf-gangliotetraosylceramide (SO_3_-3Galß3GalNAcß4Galß4Glcß1Cer; lane 5) or cholesterol-sulfate (not shown) occurred.

**Table 2:** Summary of glycosphingolipid binding specificities of F4ab, F4ac and F4ad fimbriae.

No.	Trivial name	Structure	F4ab^a^	F4ac^a^	F4ad^a^
*I. Chicken erythrocyte glycosphingolipids*					
1.	Galactosylceramide	Galß1Cer	+++	+++	−
2.		GalNAcα3GalNAcß3Galß4Glcß1Cer	+++	+++	+++
3.		GalNAcα3GalNAcß3Galß4GlcNAcß3Galß4Glcß1Cer	+++	+++	+++
*II. Porcine intestinal glycosphingolipids*					
1.	Galactosylceramide	Galß1Cer	+++	+++	−
2.	Sulfatide	SO_3_-3Galß1Cer	+++	+	−
3.	Sulf-lactosylceramide	SO_3_-3Galß4Glcß1Cer	+++	−	−
4.	Globotriaosylceramide (t18:0-h24:0)	Galα4Galß4Glcß1Cer	+++	−	−
*III. Reference glycosphingolipids*					
1.	Lactosylceramide (t18:0-h16:0-h24:0)	Galß4Glcß1Cer	+++	+++	+++
2.	Galabiaosylceramide	Galα4Galß1Cer	+++	−	−
3.	Isoglobotriaosylceramide	Galα3Galß4Glcß1Cer	−	−	+
4.	Gangliotriaosylceramide	GalNAcß4Galß4Glcß1Cer	−	−	+++
5.	Gangliotetraosylceramide	Galß3GalNAcß4Galß4Glcß1Cer	−	−	+++
6.	Neolactotetraosylceramide	Galß4GlcNAcß3Galß4Glcß1Cer	−	−	+

a F4ab denotes bindings obtained with both F4ab fimbriae and F4ab-fimbriated *E. coli*, F4ac bindings obtained with both F4ac fimbriae and F4ac-fimbriated *E. coli*, and F4ad bindings obtained with both F4ad fimbriae and F4ad-fimbriated *E. coli*.

Binding is defined as follows: +++ denotes an intense and highly reproducible staining when 4 µg of the glycosphingolipid was applied on the thin-layer chromatogram, + denotes an occasional staining,while − denotes no binding even at 4 µg.

Thus, the F4ab-binding acid glycosphingolipids of piglet small intestinal epithelium were identified as sulfatide and sulf-lactosylceramide. When using reference glycosphingolipids, the F4ab fimbriae bound to sulfatide with both sphingosine and phytosphingosine long-chain bases, and both hydroxy and non-hydroxy fatty acids, *i.e.* the ceramide composition did not influence the binding.

### Isolation and characterization of the F4ab/F4ac fimbriae binding monoglycosylceramide from pig small intestinal mucosa

The binding-active monoglycosylceramide was isolated by HPLC of the total non-acid glycosphingolipid fraction from adult pig small intestinal mucosa, and the preparative procedure was monitored by binding of radiolabeled F4ab and F4ac fimbriae on thin-layer chromatograms. Pooling of the F4ab/F4ac binding monoglycosylceramide fractions yielded 5.2 mg (denoted fraction P:mono).

**Figure 8:**
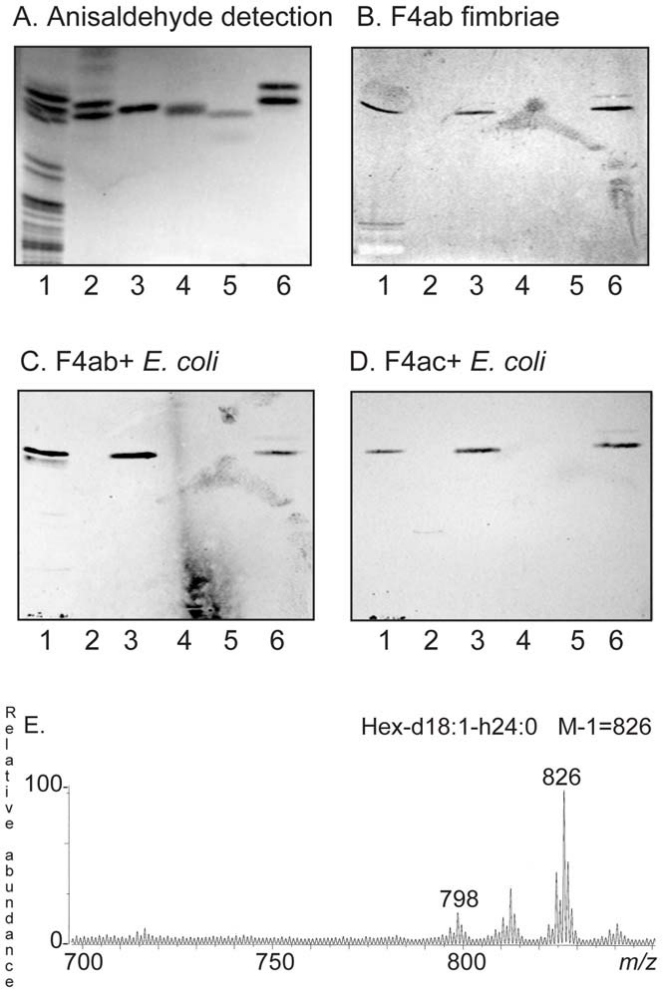
Characterization of the F4ab and F4ac binding monoglycosylceramides from porcine small intestinal mucosa. Chemical detection by anisaldehyde (A), and autoradiograms obtained by binding of ^125^I-labeled F4ab fimbriae (B), ^35^S-labeled F4ab-expressing *E. coli* (C), and F4ac-expressing *E. coli* (D). The glycosphingolipids were separated using chloroform/methanol/water 65∶25∶4 (by volume) as solvent system. The lanes were: Lane 1, non-acid glycosphingolipids of adult pig 1 small intestinal mucosa, 40 µg; Lanes 2–5, monoglycosylceramides isolated from adult pig small intestinal mucosa, 4 µg/lane; Lane 6, reference galactosylceramide (Galß1Cer) with d18:1-h18:0-h24:0 ceramide, 4 µg. (E) Negative ion FAB mass spectrum of fraction P:mono from adult pig small intestinal mucosa. Above the spectrum is an interpretation formula representing the species with d18:1-h24:0 ceramide. The analysis was done as described in the “Materials and Methods” section.

Characterization of fraction P:mono identified galactosylceramide (Galß1Cer) with sphingosine and hydroxy 24:0 fatty acid as the binding-active component. This conclusion is based on the following observations:

*I*) On thin-layer chromatograms the binding-active monoglycosylceramide migrated as a distinct band at the lower margin of the monoglycosylceramide region (Fig. 8B–D, lane 3).
*II*) The negative ion FAB mass spectrum of fraction P:mono (Fig. 8E) had a major molecular ion at *m/z* 826 identifying a monohexosylceramide with sphingosine and hydroxy 24:0 fatty acid. A major ceramide ion at *m/z* 664, obtained by elimination of the carbohydrate unit, was also present (not shown). Thus, the glycosphingolipid was identified as a monohexosylceramide with sphingosine and hydroxy 24:0 fatty acid.
*IV*) The proton NMR spectrum of fraction P:mono (data not shown) revealed two anomeric proton resonances: one belonging to Galß1Cer at 4.066 ppm (∼60%), a shift consistent with the presence of hydroxy fatty acid, and the other belonging to Glcß1Cer with non-hydroxy fatty acids at 4.113 ppm (∼40%) [22]. Both species were found to contain a sphingosine base in line with mass spectrometry data.

Using the same techniques the non-binding monoglycosylceramide fractions displayed in Fig. 8, lanes 2, 4 and 5 were characterized as glucosylceramide (Glcß1Cer) with mainly sphingosine and hydroxy 24:0 fatty acid, glucosylceramide with sphingosine and hydroxy 16:0 fatty acid and phytosphingosine with hydroxy 24:1 fatty acid together with galactosylceramide with phytosphingosine with hydroxy 16:0-24:0 fatty acids, respectively (data not shown).

Thus, of the monoglycosylceramides from pig intestine the F4ab- and F4ac-fimbriae bound to galactosylceramide with a distinct preference for the species with sphingosine and hydroxy 24:0 fatty acid.

### Isolation and characterization of the F4ab fimbriae binding non-acid triglycosylceramide of pig small intestinal mucosa

**Figure 9:**
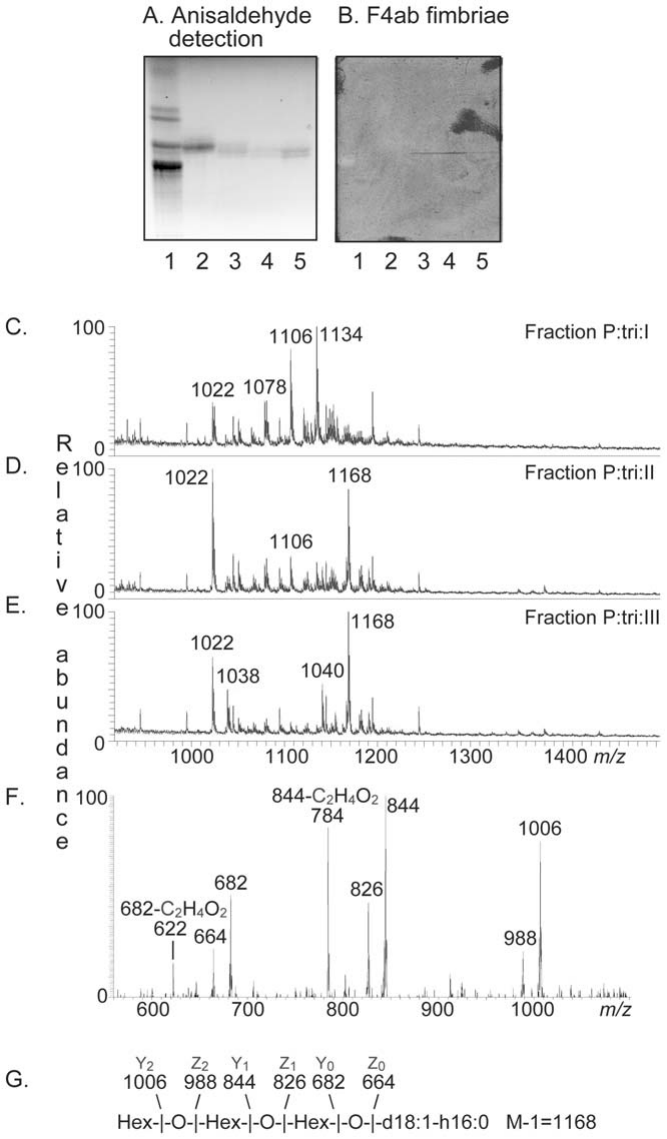
Characterization of the F4ab fimbriae binding triglycosylceramides from porcine small intestinal mucosa. Chemical detection by anisaldehyde (A), and autoradiogram obtained by binding of ^125^I-labeled F4ab fimbriae (B). The lanes were: Lane 1, non-acid glycosphingolipids of human erythrocytes, 40 µg; Lanes 2–5, fractions P:tri:I, P:tri:II, P:tri:III and P:tri:IV, respectively, from pig small intestinal epithelium, 4 µg/lane. (C) ESI mass spectrum of fraction P:tri:I from pig small intestinal epithelium. (D) ESI mass spectrum of fraction P:tri:II from pig small intestinal epithelium. (E) ESI mass spectrum of fraction P:tri:III from pig small intestinal epithelium. (F) MS^2^ spectrum of the [M-H]^−^ ion at *m/z* 1168 of fraction P:tri:III. (G) Interpretation formula representing the species with t18:0-h24:0 ceramide.

The F4ab binding triglycosylceramide was isolated by chromatography on an Iatrobeads column, and the fractions obtained were tested for F4ab binding activity using ^125^I-labeled F4ab fimbriae. The fractions obtained were pooled into four fractions according to thin-layer chromatographic resolution and binding of F4ab fimbriae (Fig. 9A, lanes 2–5). The fraction designated fraction P:tri:I (1.7 mg; lane 2) had no F4ab binding activity. Fractions P:tri:II (0.9 mg; lane 3) and P:tri:III (1.0 mg, lane 4) were recognized by the F4ab fimbriae, while fractions P:tri: IV (0.3 mg; lane 5) was non-binding.

Structural characterization of fraction P:tri:III demonstrated that the F4ab binding glycosphingolipid was globotriaosylceramide (Galα4Galß4Glcß1Cer) with phytosphingosine and hydroxy 24:0 fatty acid. This conclusion is based on the following observations:

*I*) The binding-active compound migrated in the triglycosylceramide region on thin-layer chromatograms (Fig. 6, lanes 1 and 3).
*II*) Fractions P:tri:I, P:tri: II and P:tri:III were analyzed by LC-ESI/MS using polyamine columns (to be published separately). Thereby, the non-binding fraction P:tri:I gave a series of [M-H^+^]^−^ ions at *m/z* 1022, *m/z* 1078, *m/z* 1106, and *m/z* 1134, corresponding to a glyco-sphingolipid with three Hex and sphingosine with non-hydroxy 16:0, 20:0, 22:0 and 24:0 fatty acids, respectively (Fig. 9C). A [M-H^+^]^−^ ion at *m/z* 1022, indicating a trihexosylceramide with sphingosine with non-hydroxy 16:0 fatty acid, was also present in the mass spectra of fractions P:tri: II and P:tri:III (Fig. 9D and E). However, these F4ab binding fractions also had a major peak at *m/z* 1168 indicating a glycosphingolipid with three Hex and phytosphingosine and hydroxy 24:0 fatty acid. This was confirmed by the series of fragment ions at *m/z* 1006 (Y_2_; 1168-Hex), *m/z* 844 (Y_1_; 1168-Hex-Hex) and *m/z* 682 (Y_0_; 1168-Hex-Hex-Hex) obtained by MS^2^ of the ion at *m/z* 1168 (Fig. 9F).
*III*) The proton NMR spectrum of the F4ab-fimbriae binding fraction P:tri:III (not shown) revealed a single anomeric signal at 4.78 ppm (Galα4), several overlapping signals centered around 4.26 ppm (Galß4) and three overlapping signals in the range 4.19–4.21 ppm (Glcß1), respectively, readily identifying this compound as globotriaosylceramide (Galα4Galß4Glcß1Cer) through comparison with previously published spectra [23].

**Figure 10:**
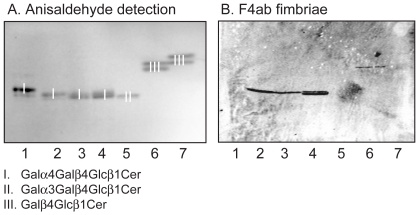
Binding of F4ab fimbriae to non-acid reference glycosphingolipids. Chemical detection by anisaldehyde (A), and autoradiogram obtained by binding of ^125^I-labeled F4ab fimbriae (B). The lanes were: Lane 1, globotriaosylceramide (Galα4Galß4Glcß1Cer) of human erythrocytes with d18:1-16:0 and d18:1-24:0 ceramide, 4 µg; Lane 2, globotriaosylceramide of rat intestine with t18:0-20:0-24:0 and t18:0-h22:0-24:0 ceramide, 2 µg; Lane 3, globotriaosylceramide of human meconium with t18:0-22:0-24:0 ceramide, 2 µg; Lane 4, globotriaosylceramide of human kidney with d18:1-h16:0 and t18:0-h22:0-h24:0 ceramide, 2 µg; Lane 5, isoglobotriaosylceramide (Galα3Galß4Glcß1Cer) of cat intestine with t18:0-h22:0-h24:0 ceramide, 2 µg; Lane 6, lactosylceramide (Galß4Glcß1Cer) of dog intestine with t18:0-h16:0-h24:0 ceramide, 4 µg; Lane 7, lactosylceramide of human neutrophils with d18:1-16:0 and d18:1-24:1 ceramide, 4 µg.

The binding of F4ab fimbriae to a panel of reference glycosphingolipids related to globotriaosylceramide was thereafter evaluated, in order to further investigate the requirements for the globotriaosylceramide binding by these fimbriae (Fig. 10). Here, the F4ab fimbriae bound to globotriaosylceramide with phytosphingosine and hydroxy or non-hydroxy fatty acids (lanes 2–4), and to lactosylceramide with phytosphingosine and hydroxy fatty acids (lane 6). However, isoglobotriaosylceramide (lane 5) was not recognized although it had phytosphingosine and hydroxy fatty acids, and no binding to globotriaosylceramide or lactosylceramide with sphingosine and non-hydroxy fatty acids (lane 1 and lane 7) occurred.

### Binding of F4 fimbriae and F4-fimbriated *E. coli* to reference glycosphingolipids

**Figure 11:**
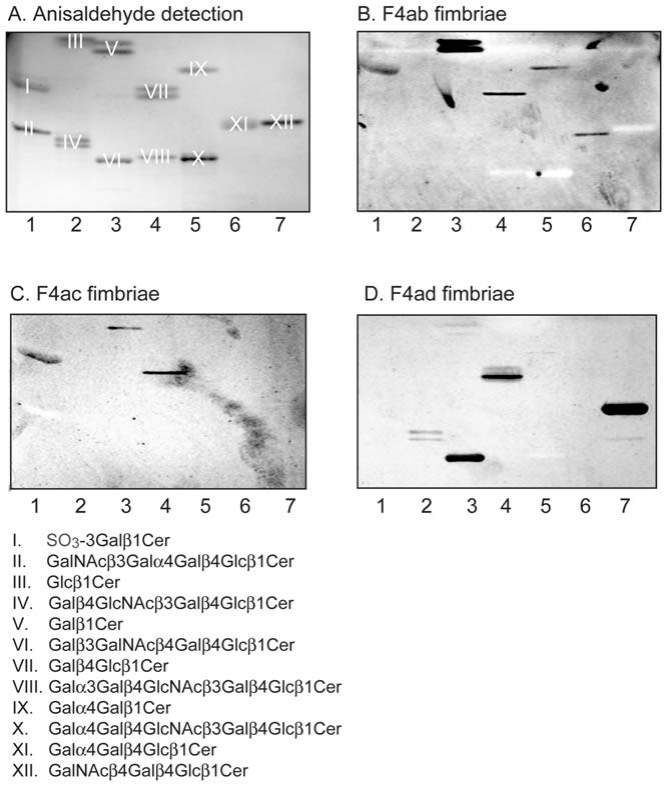
Binding of F4 fimbriae to reference glycosphingolipids. Chemical detection by anisaldehyde (A), and autoradiograms obtained by binding of ^125^I-labeled F4ab fimbriae (B), F4ac fimbriae (C), and F4ad fimbriae (D). The lanes were: Lane 1, sulfatide (SO_3_-3Galß1Cer) of human intestine with t18:0-h24:0 ceramide, 4 µg, and globotetraosylceramide (GalNAcß3Galα4Galß4Glcß1Cer) of human erythrocytes with d18:1-16:0-24:0 ceramide, 4 µg; Lane 2, glucosylceramide (Glcß1Cer) of porcine kidney with d18:1/t18:0-16:0-24:0 ceramide, 4 µg, and neolactotetraosylceramide (Galß4GlcNAcß3Galß4Glcß1Cer) of human neutrophils with d18:1-16:0 and 24:1 ceramide, 4 µg; Lane 3, galactosylceramide (Galß1Cer) of bovine brain from Sigma-Aldrich with d18:1-h18:0-h24:0 ceramide, 4 µg, and gangliotetraosylceramide (Galß3GalNAcß4Galß4Glcß1Cer) of mouse intestine with t18:0-h16:0 and h24:0 ceramide, 4 µg; Lane 4, lactosylceramide (Galß4Glcß1Cer) of dog intestine with t18:0-h16:0-h24:0 ceramide, 4 µg, and B5 pentaglycosylceramide (Galα3Galß4GlcNAcß3Galß4Glcß1Cer) of rabbit erythrocytes with d18:1-16:0 and 24:0 ceramide, 4 µg; Lane 5, galabiaosylceramide (Galα4Galß1Cer) (synthetic) with d18:1-16:0-18:0 ceramide, 4 µg, and P1 pentaglycosylceramide (Galα4Galß4GlcNAcß3Galß4Glcß1Cer) of human erythrocytes with d18:1-16:0 and 24:0 ceramide, 4 µg; Lane 6, globotriaosylceramide (Galα4Galß4Glcß1Cer) of rat intestine with t18:0-h22:0-h24:0 ceramide, 4 µg; Lane 7, gangliotriaosylceramide (GalNAcß4Galß4Glcß1Cer) of guinea pig erythrocytes with d18:1-16:0 and 24:0 ceramide, 4 µg. The glycosphingolipids visualized with anisaldehyde in (A) are marked with Roman numbers, and the corresponding glycosphingolipid structures are given below the chromatograms.

**Figure 12:**
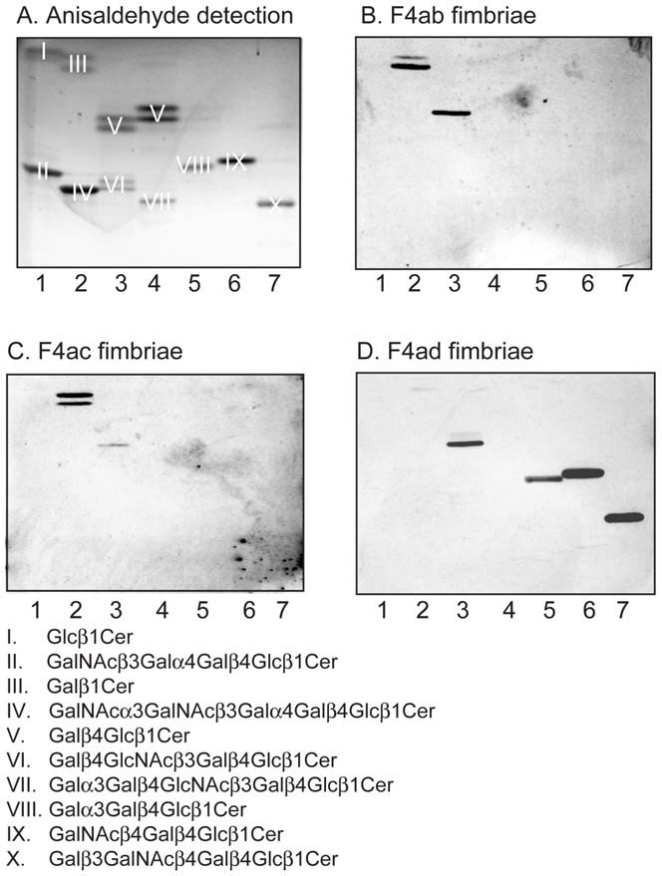
Binding of F4 fimbriae to reference glycosphingolipids. Chemical detection by anisaldehyde (A), and autoradiograms obtained by binding of ^125^I-labeled F4ab fimbriae (B), F4ac fimbriae (C), and F4ad fimbriae (D). The lanes were: Lane 1, glucosylceramide (Glcß1Cer) of porcine kidney with d18:1/t18:0-16:0-24:0 ceramide, 4 µg, and globotetraosylceramide (GalNAcß3Galα4Galß4Glcß1Cer) of human erythrocytes with d18:1-16:0-24:0 ceramide, 4 µg; Lane 2, galactosylceramide (Galß1Cer) of bovine brain from Sigma-Aldrich with d18:1-h18:0-h24:0 ceramide, 4 µg, and Forssman pentaglycosylceramide (GalNAcα3GalNAcß3Galα4Galß4Glcß1Cer) of dog intestine with d18:1-16:0 and 24:0 ceramide, 4 µg; Lane 3, lactosylceramide (Galß4Glcß1Cer) with t18:0-h16:0-h24:0 ceramide of dog intestine, 4 µg, and neolactotetraosylceramide (Galß4GlcNAcß3Galß4Glcß1Cer) of human neutrophils d18:1-16:0 and 24:1 ceramide, 4 µg; Lane 4, lactosylceramide (Galß4Glcß1Cer) with d18:1-16:0-24:1 ceramide of human neutrophils, 4 µg, and B5 pentaglycosylceramide (Galα3Galß4GlcNAcß3Galß4Glcß1Cer) of rabbit erythrocytes with d18:1-16:0 and 24:0 ceramide, 4 µg; Lane 5, isoglobotriaosylceramide (Galα3Galß4Glcß1Cer) of cat intestine with t18:0-h22:0 and h24:0 ceramide, 4 µg; Lane 6, gangliotriaosylceramide (GalNAcß4Galß4Glcß1Cer) of guinea pig erythrocytes with d18:1-16:0 and 24:0 ceramide, 4 µg; Lane 7, gangliotetraosylceramide (Galß3GalNAcß4Galß4Glcß1Cer) of mouse intestine with t18:0-h16:0 and h24:0 ceramide, 4 µg. The glycosphingolipids visualized with anisaldehyde in (A) are marked with Roman numbers, and the corresponding glycosphingolipid structures are given below the chromatograms.

To further investigate the structural requirements for F4 fimbriae glycosphingolipid recognition, the binding of F4-expressing *E. coli*, and F4 fimbriae, to a number of reference glycosphingolipids related to the binding-active compounds was next examined. The results are exemplified in Figs. 11 and 12, and summarized in Table S1. Here, lactosylceramide (Galß4Glcß1Cer; Fig. 11, lane 4, upper band, and Fig. 12, lane 3, upper band) with phytosphingosine and hydroxy fatty acid was recognized by all three subtypes of F4. However, for the remaining binding-active compounds, the recognition profiles differed between the F4 variants. Galactosylceramide (Galß1Cer; Fig. 11, lane 3, upper band, and Fig. 12, lane 2, upper band) was the preferred ligand for F4ac fimbriae and F4ac-expressing bacteria, and binding to other glycosphingolipids, as *e.g.* sulfatide (SO_3_-Galß1Cer; Fig. 11, lane 1, upper band), by the F4ac subtype occurred very occasionally. Galactosylceramide was also recognized by the F4ab fimbriae and F4ab-fimbriated bacteria. The F4ab subtype also bound to sulfatide (Fig. 11, lane 1, upper band) and to globotriaosylceramide (Galα4Galß4Glcß1Cer; Fig. 11, lane 6). In addition, galabiaosylceramide (Galα4Galß1Cer; Fig. 11, lane 5, upper band) was recognized by F4ab. Finally, the most divergent subtype was the F4ad, where both the fimbriae and the bacterial cells bound to gangliotriaosylceramide (GalNAcß4Galß4Glcß1Cer; Fig. 11, lane 7, and Fig. 12, lane 6) and gangliotetraosylceramide (Galß3GalNAcß4Galß4Glcß1Cer; Fig. 11, lane 3, lower band, and Fig. 12, lane 7), and occasionally to isoglobotriaosylceramide (Galα3Galß4Glcß1Cer; Fig. 12, lane 5) and neolactotetraosylceramide (Galß4GlcNAcß3Galß4Glcß1Cer; Fig. 11, lane 2, lower band).

### Glycosphingolipid recognition of F4ab deletion mutant fimbriae

To evaluate the relative roles of different F4ab subunits in the glycosphingolipid interaction, fimbriae were isolated from recombinant bacteria expressing F4ab fimbriae with deletions of FaeH, FaeI and FaeJ. In glycosphingolipid binding assays, the binding patterns obtained with these deletion mutant fimbriae were identical to that of the native F4ab fimbriae (Figure S4). In addition, bacteria expressing F4ab fimbriae with deletions of FaeH, FaeI and FaeJ bound in the same manner as the F4ab-positive wild type *E. coli* (Fig. S4). Thus, the minor F4ab subunits FaeH, FaeI and FaeJ are not involved in F4ab glycosphingolipid recognition.

## Discussion

Microbes often utilize cell surface glycoconjugates for target cell recognition, and the binding of bacterial pathogens to host cell glycoconjugates can be mediated by both fimbrial and non-fimbrial adhesins. Here we have investigated the glycosphingolipid recognition of F4 fimbriae, a major virulence factor of enterotoxigenic *E. coli* causing neonatal and post-weaning diarrhea. The glycosphingolipid binding preferences of the three F4 variants are summarized in Table 2.

In the initial studies using a panel of erythrocyte glycosphingolipids from different species we found a distinct binding of all three F4 variants to three chicken erythrocyte non-acid glycosphingolipids, migrating as mono-, tetra- and hexaglycosylceramides. Previous studies of chicken erythrocyte glycosphingolipids have demonstrated the presence of galactosylceramide, lactosylceramide and the Forssman pentaglycosylceramide in this source [15]. The binding of F4ab and F4ac to reference galactosylceramide, and galactosylceramide isolated from porcine intestine, suggested that the binding-active monoglycosylceramide from chicken erythrocytes was galactosylceramide. It should here be noted that no binding of the F4ad fimbriae to galactosylceramide occurred.

The F4ab/ac/ad-binding tetra- and hexaglycosylceramides of chicken erythrocytes were characterized by mass spectrometry and proton NMR as GalNAcα3GalNAcß3Galß4Glcß1Cer and GalNAcα3GalNAcß3Galß4GlcNAcß3Galß4Glcß1Cer, both of which are novel glycosphingolipid structures. GalNAcα3GalNAcα1-O-S/T is the mucin type 5 core structure. However, in glycosphingolipids the GalNAcα3GalNAc moiety has previously only been characterized in the Forssman (GalNAcα3GalNAcß3Galα4Galß4Glcß1Cer) and the iso-Forssman (GalNAcα3GalNAcß3Galα3Galß4Glcß1Cer) pentaglycosylceramides [24], [25]. No binding of the three F4 variants to the Forssman pentaglycosylceramide was obtained, suggesting that the binding epitope of the chicken tetra- and hexaglycosylceramides was the GalNAcα3GalNAcß3Galß moiety.

When using the erythrocyte panel a binding of F4ad fimbriae and F4ad-fimbriated bacteria, but not the F4ab or F4ac variants, to gangliotriaosylceramide (GalNAcß4Galß4Glcß1Cer), the major non-acid glycosphingolipid of guinea pig erythrocytes was also obtained.

However, there was no correlation between the hemagglutination patterns of the F4 variants and the glycosphingolipid bindings obtained, suggesting that the glycosphingolipids are not involved in the hemagglutination process.

Next, the binding of the F4 variants to glycosphingolipids from the small intestinal epithelium of neonatal and adult pigs was evaluated. Here, no binding of the F4ad fimbriae or F4ad-fimbriated *E. coli* to the acid or non-acid glycosphingolipids of pig intestine was obtained. However, for F4ab and F4ac two distinct binding patterns were observed. The F4ac fimbriae and the F4ac-expressing *E. coli* selectively bound to galactosylceramide with sphingosine and hydroxy 24:0 fatty acid. This compound was also recognized by the F4ab fimbriae and the F4ab-fimbriated bacteria. Binding of F4ab fimbriae to galactosylceramide in piglet ileal mucus has been described previously [26]. In addition, we found that the F4ab fimbriae and F4ab-fimbriated bacteria also bound to sulfatide, sulf-lactosylceramide and globotriaosylceramide with phytosphingosine and hydroxy or non-hydroxy fatty acids.

Finally, when the binding to reference glycosphingolipids from other sources was tested all three serotypes bound to lactosylceramide (Galß4Glcß1Cer) with hydroxy ceramide. F4ab also recognized galabiaosylceramide (Galα4Galß1Cer), in line with the binding to porcine intestinal globotriaosylceramide (Galα4Galß4Glcß1Cer). In addition, a selective binding of the F4ad fimbriae and the F4ad-fimbriated *E. coli*, but not the F4ab or F4ac subtypes, to reference gangliotriaosylceramide (GalNAcß4Galß4Glcß1Cer) and gangliotetraosylceramide (Galß3GalNAcß4Galß4Glcß1Cer) was observed, and occasionally F4ad also bound to isoglobotriaosylceramide (Galα3Galß4Glcß1Cer) and neolactotetraosylceramide (Galß4GlcNAcß3Galß4Glcß1Cer).

The major subunit FaeG is considered to be the adhesive subunit, since sequence comparison of the genes encoding the minor and major fimbrial subunits of the F4ab and F4ac variants showed that the differences between these variants are confined to the *faeG* gene [8]. This was supported by the fact that removal of all minor fimbrial subunits by treatment of the F4 fimbriae with 2 M urea at 55°C did not affect the adhesive properties of the fimbriae [7].

In general, the F4ab and F4ac variants showed more similarities in their glycosphingolipid recognition patterns compared to the F4ad variant (Table S1 and Table 2). Comparative analysis of the FaeG sequences from various F4ab, F4ac and F4ad antigenic variant strains revealed that the amino acid sequences of the F4ab and F4ac variants had a higher degree of homology (92%), compared to the homology between the F4ad and F4ac (88%) [27]. Within each F4 serotype, 96–100% homology in the FaeG amino acid sequence was observed. Interestingly, an unusually large carbohydrate binding site, consisting of an enlarged Ig-domain with insertion of two α-helices and two ß-strands, is found in the crystal structure of the F4ad variant of FaeG [28].

The repertoire of glycosphingolipids recognized by the F4ab fimbriae is the most diverse, since this fimbrial subtype binds to sulfatide, sulf-lactosylceramide, galactosylceramide, lactosylceramide, galabiaosylceramide, globotriaosylceramide and the two GalNAcα3GalNAcß3Galß-terminated glycosphingolipids of chicken erythrocytes. Currently it is not known if all these compounds are accomodated in the same carbohydrate binding site, and there is no direct evidence for the involvement of the FaeG subunits in the glycosphingolipid binding. However, in the glycosphingolipid binding assays with fimbriae isolated from recombinant bacteria expressing F4ab fimbriae with deletions of FaeH, FaeI or FaeJ, the binding patterns obtained were identical to that of the native F4ab fimbriae, and the bacteria expressing F4ab fimbriae with deletions of FaeH, FaeI and FaeJ bound in the same manner as the F4ab-positive wild type *E. coli*. These results suggest that the glycosphingolipid binding is not mediated by the minor F4ab fimbrial shaft subunit FaeH, or the minor FaeI and FaeJ subunits. Binding studies with ^125^I-labeled recombinant F4ac/FaeG were also attempted but gave only non-specific binding, probably due to the low stability of this protein [29].

A switch in porcine brush border binding specificity was obtained with *E. coli* expressing F4ac/F4ad chimeric FaeG subunits, produced by reciprocal exchange of amino acids 125 to 163 [30]. It would be highly interesting to examine if a parallel change in glycosphingolipid binding specificity occurs with bacteria expressing these chimeric FaeG subunits.

The reason for the discrepancy between the results from the previous F4 glycosphingo-lipid binding studies [11], [12] and our results is unclear. The focus of our study was the F4-binding glycosphingolipids present in erythrocytes and the epithelial cells of pig intestine. Payne *et al.* studied the binding of F4ab fimbriated *E. coli* to reference glycosphingolipids from various sources on thin-layer plates, along with binding of F4ab fimbriae to reference glycosphingolipids in microtiter wells [11]. However, some of the F4ab-binding glycosphingolipids of porcine intestine, as *e.g.* sulfatide and sulf-lactosylceramide, were not included in that study, and relatively high amounts (10 µg) of glycosphingolipids were used in the chromatogram binding experiments. The main difference between our study and the study by Grange *et al.* was that biotinylated F4 fimbriae were used in the latter study [12].

The repertoire of porcine intestinal glycosphingolipids recognized by the F4 fimbriae has, in different combinations, been reported as putative receptors for a large number of other microbes and microbial proteins. Notably, the PapG adhesin of uropathogenic P-fimbriated *E. coli* and the verotoxin family (VT-1, VT-2 and SLT-II) bind to globotriaosylceramide, with binding dependent on the Galα4Gal epitope [31]–[33]. The PapG adhesin and the pig edema disease toxin (VTE or SLT-IIv) also bind to globotetraosylceramide [31], [34], [35], not recognized by F4ab, but do not bind either galactosylceramide or sulfatide. That the Galα4Gal motif is indeed recognized by the F4ab fimbriae is shown by the parallel binding of this fimbrial subtype to galabiaosylceramide. However, for the F4ab fimbriae a substitution of the terminal Gal with a ßGalNAc in 3-position is not tolerated since globoside is non-binding.

Binding of uropathogenic F1c-fimbriated *E. coli* to galactosylceramide and globotriaosylceramide with hydroxy ceramides, but not to sulfatide, has also been reported [36].

Sulfatide recognition is also a common theme in microbial adhesion, and binding to sulfatide has been reported for *e.g.* colonization factor antigen CS6 from human ETEC, *Mycoplasma pneumoniae*, *Bordetella pertussis*, 987P-fimbriated *E. coli*, and *Helicobacter pylori* [37]–[42]. Interestingly, the subtype of the heat-stable enterotoxin of *E. coli* which is primarily associated with diarrhea in piglets (the STb subtype) also binds to sulfatide [43].

Finally, the binding pattern of F4ad, with recognition of lactosylceramide with hydroxy ceramide, isoglobotriaosylceramide, neolactotetraosylceramide, gangliotriaosylceramide and gangliotetraosylceramide, has previously been reported for several bacteria, both pathogens and members of the indigenous flora [44]. It should, however, be noted that no binding of the F4ad fimbriae and the F4ad-fimbriated *E. coli* to the porcine intestinal glycosphingolipids was obtained, suggesting that the glycosphingolipid binding of F4ad has no relevance for bacterial attachment to the porcine small intestinal epithelium.

The objective of present study was to characterize the erythrocyte glycosphingolipids, and the glycosphingolipids of porcine small intestinal epithelium, recognized by the F4ab, F4ac and F4ad fimbriae, with the ultimate goal to create a platform for synthesis of anti-adhesive substances. Candidate receptors for adherence to the epithelial cells of porcine intestine were identified as galactosylceramide, sulfatide, sulf-lactosylceramide and globotriaosylceramide for F4ab, galactosylceramide only for F4ac, while no binding of F4ad to the porcine intestinal glycosphingolipid samples occurred. Still, F4ad binding neolactotetraosylceramide has previously been identified in porcine intestinal epithelial cells [13]. Whether the presence of the glycosphingolipids recognized by the F4 variants is related to the differences in the adhesiveness of F4 subtypes to pig intestinal brush border membranes is currently investigated.

Furthermore, two candidate porcine intestinal glycoprotein receptors for the F4 fimbriae have previously been identified, *i.e.* a pair of mucin-type sialoglycoproteins (210 kDa or 240 kDa) recognized by F4ab and F4ac, and a 74-kDa transferrin glycoprotein recognized by F4ab (reviewed in [2], [45]). Characterization of the glycans of these F4 binding glycoproteins is an important next step.

## Materials and Methods

### Bacterial strains, culture conditions and labeling

The wild type F4ab-positive *E. coli* strains C585-80 (serotype O8: K87: H19:F4ab, LT+), the wild type F4ac-positive *E. coli* strain IMM01 (serotype O149:K91:F4ac, LT+, STb+) [46], and the wild type F4ad-positive *E. coli* C1360-79 (serotype O8:H10:F4ad) (15) were cultured on BHI agar plates (Oxoid, Basingstoke, Hampshire, England) at 37°C for 18 h (7). *E. coli* K-12 (C600, λ^−^ *tonA21 thr leu-6 thi-1 supE44 lacY-1* ^stable^) [47] containing the F4ab-encoding plasmid pDB88-8, and the mutant derivatives of pDB88-8, were grown on BHI agar plates, supplemented with ampicillin (100 µg/ml) at 37°C over night. The FaeH- mutant strain contains a stop codon in *faeH*, whereas the FaeI mutant strain contains a deletion in *faeI*, and the FaeJ mutant strain contains a frameshift deletion in *faeJ*. The resulting plasmids were named pDB88-141 (FaeH- mutant), pDB88-85 (FaeI- mutant) and pDB88-84 (FaeJ- mutant) [7]. After growing, the bacteria were harvested by centrifugation and suspended in PBS (phosphate-buffered saline, pH 7.3). The concentration of bacteria in the suspensions were determined by measuring the optical density at 660 nm (*A*_660_). An optical density of 1 equals 10^9^ bacteria per milliliter, as determined by counting colony forming units.

For metabolic labeling, the culture plates were supplemented with 10 µl ^35^S-methionine (400 µCi; Amersham Pharmacia Biotech). Bacteria were harvested, washed three times in PBS, and resuspended in PBS containing 2% (w/v) bovine serum albumin (MP Biomedicals, LLC, Illkirch, France) 0.1% (w/v) NaN_3_ and 0.1% (w/v) Tween 20 (BSA/PBS/TWEEN) to a bacterial density of 1×10^8^ colony forming units/ml. The specific activity of bacterial suspensions was approximately 1 cpm per 100 bacteria.

### Fimbrial preparations

The F4ab-, F4ac- and F4ad wild type fimbriae and F4ab mutant fimbriae were purified as described by Van den Broeck *et al.* [48]. In short, the wild type F4 positive *E. coli* were grown in tryptone soy broth (DIFCO Laboratories, Biotrading, Bierbeek, Belgium) at 37°C for 18 h while shaking at 85 rpm. The FaeH-, FaeI- and FaeJ-mutant strains were grown in tryptone soy broth, supplemented with ampicillin (100 µg/ml) [7]. Bacteria were harvested by centrifugation (3500 rpm, 30 min, 4°C), washed and suspended in PBS. Subsequently, F4 fimbriae were isolated by homogenizing the bacterial suspension using an Ultra Turrax (Janke & Kunkel, IKA Labortechnik, Staufen, Germany) at 24,000 rpm for 15 min keeping the suspension on ice. Next, the bacteria were pelleted by centrifugation for 20 min at 10,000×g at 4°C, and the supernatant was further purified by centrifugation for 40 min at 20,000×g at 4°C. The solubilized fimbriae were subsequently precipitated with 40% ammonium sulfate. After centrifugation, the pellet was dissolved and dialyzed overnight against PBS. The protein concentration of the isolated fimbriae was determined using the bicinchoninic acid protein assay kit (Sigma-Aldrich, Bornem, Belgium).

### ^125^I-labeling

Aliquots of 100 µg of protein were labeled with ^125^I, using Na^125^I (100 mCi/ml; Amersham Pharmacia Biotech, Little Chalfont, U.K.), according to the IODO-GEN protocol of the manufacturer (Pierce, Rockford, IL), giving approximately 2×10^3^ cpm/µg protein.

### Reference glycosphingolipids

Total acid and non-acid glycosphingolipid fractions were isolated as described [49]. Individual glycosphingolipids were isolated by repeated chromatography on silicic acid columns and by HPLC, and identified by mass spectrometry [50] and ^1^H-NMR spectroscopy [18]. Galactosylceramide (Galß1Cer) of bovine brain with d18:1-h18:0-h24:0 ceramide was purchased from Sigma-Aldrich, St. Louis, MO. Synthetic galabiaosylceramide (Galα4Galß1Cer) was a kind gift from late Dr. Göran Magnusson, Lund University, Sweden.

### Thin-layer chromatography

Thin-layer chromatography was done on aluminum- or glass-backed silica gel 60 high performance thin-layer chromatography plates (Merck, Darmstadt, Germany). Glycosphingolipid mixtures (10–80 µg) or pure glycosphingolipids (0.5–4 µg) were applied to the plates, and if not otherwise stated, eluted with chloroform/methanol/water (60∶35∶8, by volume). Chemical detection was done with anisaldehyde [51].

### Chromatogram binding assay

Binding of radiolabeled fimbriae and bacteria to glycosphingolipids on thin-layer chromatograms was done as described previously [37]. Dried chromatograms were dipped in diethylether/*n*-hexane (1∶5 v/v) containing 0.5% (w/v) polyisobutylmethacrylate for 1 min. To diminish background binding the chromatograms were blocked with BSA/PBS/TWEEN for 2 h at room temperature. Then the plates were incubated with ^125^I-labeled fimbriae (1–5×10^6^ cpm/ml) or ^35^S-labeled bacteria (1–5×10^6^ cpm/ml) diluted in BSA/PBS/TWEEN for another 2 h at room temperature. After washing six times with PBS, and drying, the thin-layer plates were autoradiographed for 12 h using XAR-5 x-ray films (Eastman Kodak, Rochester, NY).

### Isolation of the F4 fimbriae binding slow-migrating non-acid glycosphingolipids from chicken erythrocytes

Acid and non-acid glycosphingolipids were isolated from chicken erythrocytes by standard methods [49]. Briefly, the erythrocytes were lyophilized and then extracted in two steps in a Soxhlet apparatus with chloroform and methanol (2∶1 and 1∶9, by volume, respectively). The material obtained was subjected to mild alkaline hydrolysis and dialysis, followed by separation on a silicic acid column. Acid and non-acid glycosphingolipid fractions were obtained by chromatography on a DEAE-cellulose column. In order to separate the non-acid glycolipids from alkali-stable phospholipids, this fraction was acetylated and separated on a second silicic acid column, followed by deacetylation and dialysis. Final purifications were done by chromatographies on DEAE-cellulose and silicic acid columns.

Part of the non-acid glycosphingolipids (8.4 mg) were separated on a 10 g Iatrobeads (Iatrobeads 6RS-8060; Iatron Laboratories, Tokyo) column, eluted with chloroform/methanol/water 65∶25∶4 (by volume), 32×0.5 ml, followed by chloroform/methanol/water 60∶35∶8 (by volume), 66×0.5 ml, and finally chloroform/methanol/water 40∶40∶12 (by volume), 15 ml. The fractions obtained were tested for binding of F4ab and F4ad fimbriae using the chromatogram binding assay. The F4-binding glycosphingolipid migrating in the tetraglycosylceramide region was eluted in fractions 65–71. Pooling of these fractions yielded 0.9 mg (designated fraction C:tetra-I). The binding-active compound migrating in the hexaglycosphingolipid region eluted in fractions 91–97, and after pooling of these fractions less than 0.1 mg was obtained (designated fraction C:hexa). Proton NMR revealed that fraction C:tetra-I was a mixture of four glycosphingolipids. This fraction was therefore further separated on a 1 g Iatrobeads column, eluted with chloroform/methanol/water 60∶35∶8 (by volume), 10×0.5 ml. Pooling of the F4-binding fractions gave 0.2 mg (designated fraction C:tetra-II).

### Isolation of the F4ab/F4ac fimbriae binding non-acid monoglycosylceramide from pig small intestinal mucosa

Acid and non-acid glycosphingolipids were isolated from mucosal scrapings from the small intestine of an adult pig as described [49]. Part of the non-acid glycosphingolipids (150 mg) was thereafter separated by HPLC on a 2.1×25 cm Kromasil 5 Silica column (particle size 5 µm; Phenomenex, Torrence, CA), eluted with a linear gradient of chloroform/methanol/water 90∶10∶1 to 60∶35∶8 (by volume) during 180 min with a flow rate of 2 ml/min. Aliquots of each 2 ml fraction were analyzed by thin-layer chromatography, and the fractions positive for anisaldehyde staining were further tested for binding of F4ab fimbriae, using the chromatogram binding assay. Glycosphingolipids migrating as monoglycosylceramides were collected in tubes 41–72, and the F4ab fimbriae binding compound was collected in tubes 62–65. Pooling of tubes 62–65 yielded 5.2 mg, and this fraction (designated fraction P:mono) was used for structural characterization.

### Isolation of the F4ab fimbriae binding non-acid triglycosylceramide from pig small intestinal mucosa

The subfractions containing glycosphingolipids migrating as diglycosylceramides and below, from the separation described above, were pooled giving 26.4 mg. This material was separated on an Iatrobeads (Iatrobeads 6RS-8060; Iatron Laboratories, Tokyo) column (10 g), eluted with chloroform/methanol/water 65∶25∶4 (by volume), 4×5 ml, followed by 36×1 ml, and finally chloroform/methanol/water 60∶35∶8 (by volume), 1×10 ml. Compounds migrating in the triglycosylceramide region were collected in fractions 14–29. The fractions were pooled into four fractions according to thin-layer chromatographic resolution and binding of F4ab fimbriae. The fraction designated fraction P:tri:I (1.7 mg) had no F4ab binding activity. Fractions P:tri:II (0.9 mg) and P:tri:III (1.0 mg) were recognized by the F4ab fimbriae, while fractions P:tri:IV (0.3 mg) was non-binding.

### Thin-layer chromatography - negative ion FAB mass spectrometry

The acid glycosphingolipid fraction from newborn piglet small intestinal mucosa (50 µg) was separated on aluminium-backed silica gel 60 HPTLC plates using chloroform/methanol/water 60∶35∶8 (by volume) as solvent system. Several thin-layer chromatograms were developed in parallel for bacterial binding, chemical staining with anisaldehyde and thin-layer chromatography - negative ion FAB mass spectrometry (TLC-FAB-MS), respectively [52]. Mass spectrometry was performed with a ZAB-2F/HF mass spectrometer (VG Analytical, Manchester, UK). For TLC-FAB-MS, a movable FAB probe (VG Analytical) was used. The thin-layer plates with separated glycosphingolipids were cut into 6 mm wide strips, and after mounting on the probe, a layer of triethanolamine (Fluka, Buchs, Switzerland) was applied using a soft roller. Negative ion FAB mass spectra were produced by Xe atoms, 8 kV. Four scans per mm of the thin-layer plate were recorded.

### Negative ion FAB mass spectrometry

Negative ion FAB mass spectra of purified glycosphingolipids were recorded on a JEOL SX-102A mass spectrometer (JEOL, Tokyo, Japan). The ions were produced by 6 keV xenon atom bombardment, using triethanolamine (Fluka, Buchs, Switzerland) as matrix, and an accelerating voltage of −10 kV.

### Endoglycoceramidase digestion and LC/MS

Endoglycoceramidase II from *Rhodococcus* spp. [53] (Takara Bio Europe S.A., Gennevilliers, France) was used for hydrolysis of glycosphingolipids. Briefly, 50 µg of the F4ab fimbriae binding non-acid glycosphingolipid fractions from chicken erythrocytes (fractions C:tetra and C:hexa) were resuspended in 100 µl 0.05 M sodium acetate buffer, pH 5.0, containing 120 µg sodium cholate, and sonicated briefly. Thereafter, 1 mU of endoglycoceramidase II was added and the mixture was incubated at 37°C for 48 h. The reaction was stopped by addition of chloroform/methanol/water to the final proportions 8∶4∶3 (by volume). The oligosaccharide-containing upper phase thus obtained was separated from detergent on a Sep-Pak QMA cartridge (Waters, Milford, MA). The eluant containing the oligosaccharides was dried under nitrogen and under vacuum.

The glycosphingolipid-derived oligosaccharides were analyzed by capillary-LC/MS and MS/MS as described [16]. In brief, the oligosaccharides were separated on a column (200×0.180 mm) packed in-house with 5 µm porous graphite particles (Hypercarb, Thermo Scientific), and eluted with an acetonitrile gradient (A: 8 mM ammonium bicarbonate; B: 100% acetonitrile). The saccharides were analyzed in the negative ion mode on an LTQ linear quadrupole ion trap mass spectrometer (Thermo Electron, San José, CA).

### ESI/MS and ESI/MS/MS of native glycosphingolipids

The glycosphingolipids (dissolved in methanol) were analyzed on an LTQ linear quadrupole ion trap mass spectrometer by static nano-ESI/MS at −1.7 kV, using type F gold-coated needles (Micromass/Waters, Milford, MA). Full-scan (*m/z* 380–2 000, 2 microscans, maximum 100 ms, target value of 30 000) was performed, followed by data dependent MS^2^ scans (2 microscans, maximum 100 ms, target value of 10 000) with normalized collision energy of 30%, an isolation window of 3 µ, an activation q = 0.25, and an activation time of 30 ms.

### Proton NMR spectroscopy

^1^H NMR spectra were acquired on a Varian 600 MHz spectrometer at 30°C. Samples were dissolved in dimethyl sulfoxide/D_2_O (98∶2, by volume) after deuterium exchange. Two-dimensional double quantum-filtered correlated spectroscopy (DQF-COSY) spectra were recorded by the standard pulse sequence [54].

## Supporting Information

Figure S1 **Purified wild type and deletion mutant F4 fimbriae.** The protein preparations were separated by SDS-PAGE (12%), and stained by Coomassie Brilliant Blue R-250. The lanes were; Lane 1, F4ab fimbriae with deletion of FaeH, 5 µg; Lane 2, F4ab fimbriae with deletion of FaeI, 5 µg; Lane 3, F4ab fimbriae with deletion of FaeJ, 5 µg; Lane 4, wild type F4ab fimbriae, 5 µg; Lane 5, wild type F4ac fimbriae, 5 µg; Lane 6, wild type F4ad fimbriae, 5 µg; Lane 7, molecular weight marker (kDa).
(TIFF)

Figure S2 **ESI/MS/MS of the native fraction C:Tetra-II from chicken erythrocytes.** Above the spectrum is an interpretation formula representing the molecular species with t18:0-h16:0 ceramide.
(TIFF)

Figure S3 **TLC-FAB-MS of the acid glycosphingolipids from newborn piglet small intestinal mucosa.** (A) Thin-layer chromatogram stained with anisaldehyde. Acid glycosphingolipids (50 µg) of the epithelial cells of neonatal piglet small intestine were separated on aluminum-backed HPTLC plates using chloroform/methanol/water 60∶35∶8 (by volume) as solvent system. (B) Autoradiogram obtained by binding of ^125^I-labeled F4ab fimbriae to the acid glycosphingolipids of newborn piglet small intestine. (C–E) Reconstructed curves of selected ions of NeuGc-GM3 (C), sulfated dihexosylceramide (D) and sulfated monohexosylceramide (E), representing the successive detection of different ceramide species. TLC-FAB-MS was performed as described [52], using a ZAB-2F/HF mass spectrometer (VG Analytical, Manchester, UK). Four scans per mm of the thin-layer plate were recorded. The scanning of the thin-layer plate started at the bottom (to the left in the figure) and was run approximately 60 mm upwards, giving in total about 250 scans. After scanning approximately 40 mm of the thin-layer chromatogram, *i.e.* at the level of the more slow-migrating F4ab-binding glycosphingolipid, peaks appeared that corresponded to molecular ions of sulfated dihexosylceramide. In scans 157–164, the peak at *m/z* 956, corresponding to the species with d18:1-h16:0, was dominating, and in scan 165 the species with t18:0-16:0 (*m/z* 958) dominated. The following scans had peaks corresponding to d18:1-16:0 (*m/z* 940), d18:1-h22:0 (*m/z* 1040), and d18:1-h24:0 or t18:0-24:1 (*m/z* 1068) ceramides. At the level of the more fast-migrating F4ab-binding compound (scans 168–211) molecular weight ions of sulfated monohexosylceramide were obtained. Here, ions corresponding to sulfated monohexosylceramide with d18:1-h16:0 (*m/z* 794), d18:1-16:0 (*m/z* 778), d18:1-h22:0 (*m/z* 878), d18:1-h24:0 or t18:0-24:1 (*m/z* 906), and d18:1-24:0 (*m/z* 890) were found. Thus, the more slow-migrating F4ab-binding glycosphingolipid was tentatively identified as sulfated dihexosylceramide, while the fast-migrating F4ab-binding binding glycosphingolipid was tentatively identified as sulfated monohexosylceramide.
(TIFF)

Figure S4 **Binding of deletion mutant F4ab fimbriae to glycosphingolipids.** Thin-layer chromatograms after chemical detection by anisaldehyde (A and J), and autoradiograms obtained by binding of ^35^S-labeled native F4ab fimbriae (B), F4ab fimbriae with deletions of FaeH (C), FaeI (D) and FaeJ (E), and ^35^S-labeled *E. coli* expressing native F4ab fimbriae (F), and F4ab fimbriae with deletions of FaeH (G and K), FaeI (H and L) and FaeJ (I and M). The glycosphingolipids were separated on aluminum-backed silica gel plates, using chloroform/methanol/water (60∶35∶8, by volume) as solvent system, and the binding assays were performed as described under “Materials and Methods”. Autoradiography was for 12 h. The lanes on A-I were: Lane 1, Sulfatide (SO_3_-3Galß1Cer) with t18:0-h24:0 ceramide, 4 µg; Lane 2, Galactosylceramide (Galß1Cer) with d18:1-h18:0-h24:0 ceramide, 4 µg; Lane 3, Lactosylceramide (Galß4Glcß1Cer) with t18:0-h16:0-h24:0 ceramide, 4 µg; Lane 4, globotriaosylceramide (Galα4Galß4Glcß1Cer) with t18:0-22:0-24:0 ceramide, 4 µg; Lane 5, globotetraosylceramide (GalNAcß3Galα4Galß4Glcß1Cer) with t18:0-h16:0-h24:0 ceramide, 4 µg. The lanes on J-M were: Lane 6, Lactosylceramide (Galß4Glcß1Cer) with t18:0-h16:0-h24:0 ceramide, 4 µg; Lane 7, globotriaosylceramide (Galα4Galß4Glcß1Cer) with d18:1-16:0 and d18:1-24:0 ceramide, 4 µg; Lane 8, GalNAcα3GalNAcß3Galß4Glcß1Cer of chicken erythrocytes, 4 µg. The glycosphingolipids visualized with anisaldehyde in (A) and (J) are marked with Roman numbers, and the corresponding glycosphingolipid structures are are given to the right of the chromatograms.
(TIFF)

Table S1 **Summary of results from binding of F4 fimbriae and F4-fimbriated** ***Escherichia coli*** **to glycosphingolipids on thin-layer chromatograms.**
(DOC)

## References

[1] Fairbrother JM, Nadeau E, Gyles CL (2005). *Escherichia coli* in postweaning diarrhea in pigs: an update on bacterial types, pathogenesis, and prevention strategies.. Anim Health Res Re.

[2] Van den Broeck W, Cox E, Oudega B, Goddeeris BM (2000). The F4 fimbrial antigen of *Escherichia coli* and its receptors.. Vet Microbiol.

[3] Mol O, Oudega B (1996). Molecular and structural aspects of fimbriae biosynthesis and assembly in *Escherichia coli*.. FEMS Microbiol Rev.

[4] Oudega B, de Graaf M, de Boer L, Bakker D, Vader CE (1989). Detection and identification of FaeC as a minor component of K88 fibrillae of *Escherichia coli*.. Mol Microbiol.

[5] Valent QA, Zaal J, de Graaf FK, Oudega B (1995). Subcellular localization and topology of the K88 usher FaeD in *Escherichia coli*.. Mol Microbiol.

[6] Bakker D, Vader CE, Roosendaal B, Mooi FR, Oudega B (1991). Structure and function of periplasmic chaperone-like proteins involved in the biosynthesis of K88 and K99 fimbriae in enterotoxigenic *Escherichia coli*.. Mol Microbiol.

[7] Bakker D, Willemsen PT, Willems RH, Huisman TT, Mooi FR (1992). Identification of minor fimbrial subunits involved in biosynthesis of K88 fimbriae.. J Bacteriol.

[8] Bakker D, Willemsen PT, Simons LH, van Zijderveld FG, de Graaf FK (1992). Characterization of the antigenic and adhesive properties of FaeG, the major subunit of K88 fimbriae.. Mol Microbiol.

[9] Guinée PA, Jansen WH (1979). Behavior of *Escherichia coli* K antigens K88ab, K88ac, and K88ad in immunoelectrophoresis, double diffusion, and hemagglutination.. Infect Immun.

[10] Gaastra W, Klemm P, De Graaf FK (1983). The nucleotide sequence of the K88ad protein subunit of porcine enterotoxigenic *Escherichia coli*.. FEMS Microbiol Lett.

[11] Payne D, O'Reilly M, Williamson D (1993). The K88 fimbrial adhesin of enterotoxigenic *Escherichia coli* binds to ß1-linked galactosyl residues in glycosphingolipids.. Infect Immun.

[12] Grange PA, Mouricourt MA, Levery SB, Francis DH, Erickson AK (2002). Evaluation of receptor binding specificity of *Escherichia coli* K88 (F4) fimbrial adhesin variants using porcine serum transferrin and glycosphingolipids as model receptors.. Infect Immun.

[13] Grange PA, Erickson AK, Levery SB, Francis DH (1999). Identification of an intestinal neutral glycosphingolipid as a phenotype-specific receptor for the K88ad fimbrial adhesin of *Escherichia coli*.. Infect Immun.

[14] Seyama Y, Yamakawa T (1974). Chemical structure of glycolipids of guinea pig red blood cell membrane.. J Biochem.

[15] Shiraishi T, Uda Y (1985). Characterization of neutral sphingolipids from chicken erythrocytes.. J Lipid Res.

[16] Karlsson H, Halim A, Teneberg S (2010). Differentiation of glycosphingolipid-derived glycan structural isomers by liquid chromatography-mass spectrometry.. Glycobiology.

[17] Dabrowski J, Hanfland P, Egge H (1980). Structural analysis of glycosphingolipids by high-resolution ^1^H nuclear magnetic resonance spectroscopy.. Biochemistry.

[18] Koerner TAW, Prestegard JH, Demou PC, Yu RK (1983). High-resolution proton NMR studies of gangliosides. 1. Use of homonuclear two-dimensional spin-echo J-correlated spectroscopy for determination of residue composition and anomeric configurations.. Biochemistry.

[19] Teneberg S, Ångström J, Ljungh Å (2004). Carbohydrate recognition by enterohemorrhagic *Escherichia coli*. Characterization of a novel glycosphingolipid from cat small intestine.. Glycobiology.

[20] Holgersson J, Jovall P-Å, Breimer ME (1991). Glycosphingolipids of human large intestine: detailed structural characterization with special reference to blood group compounds and bacterial receptor structures.. J Biochem.

[21] Thorn JJ, Levery SB, Salyan MEK, Stroud MR, Cedergren B (1992). Structural characterization of x_2_ glycosphingolipid, its extended form, and its sialosyl derivatives: Accumulation associated with the rare blood group p phenotype.. Biochemistry.

[22] Dabrowski J, Egge H, Hanfland P (1980). High resolution nuclear magnetic resonance spectroscopy of glycosphingolipids. I: 360 MHz 1H and 90.5 MHz 13C NMR analysis of galactosylceramides.. Chem Phys Lipids.

[23] Poppe L, Dabrowski J, von der Lieth C-W, Koike K, Ogawa T (1990). Three-dimensional structure of the oligosaccharide terminus of globotriaosylceramide and isoglobotriaosylceramide in solution. A rotating-frame NOE study using hydroxyl groups as long-range sensors in conformational analysis by 1H-NMR spectroscopy.. Eur J Biochem.

[24] Karlsson KA, Leffler H, Samuelsson BE (1974). Characterization of the forssman glycolipid hapten of horse kidney by mass spectrometry.. J Biol Chem.

[25] Falk P, Holgersson J, Jovall PA, Karlsson KA, Strömberg N (1986). An antigen present in rat adenocarcinoma and normal colon non-epithelial stroma is a novel Forssman-like glycolipid based on isoglobotetraosylceramide.. Biochim Biophys Acta.

[26] Blomberg L, Krivan HC, Cohen PS, Conway PL (1993). Piglet ileal mucus contains protein and glycolipid (galactosylceramide) receptors specific for *Escherichia coli* K88 fimbriae.. Infect Immun.

[27] Verdonck F, Cox E, Schepers E, Imberechts H, Joensuu J (2004). Conserved regions in the sequence of the F4 (K88) fimbrial adhesin FaeG suggest a donor strand mechanism in F4 assembly.. Vet Microbiol.

[28] De Greve H, Wyns L, Bouckaert J (2007). Combining sites of bacterial fimbriae.. Curr Op Struct Biol.

[29] Verdonck F, Cox E, Van der Stede Y, Goddeeris BM (2004). Oral immunization of piglets with recombinant F4 fimbrial adhesin FaeG monomers induces a mucosal and systemic F4-specific immune response.. Vaccine.

[30] Zhang W, Fang Y, Francis DH (2009). Characterization of the binding specificity of K88ac and K88ad fimbriae of enterotoxigenic *Escherichia coli* by constructing K88ac/K88ad chimeric FaeG major subunits.. Infect Immun.

[31] Bock K, Breimer ME, Brignole A, Hansson GC, Karlsson K-A (1985). Specificity of binding of a strain of uropathogenic *Escherichia coli* to Gal alpha 1- 4Gal-containing glycosphingolipids.. J Biol Chem.

[32] Lingwood CA, Law H, Richardsson S, Petric M, Brunton JL (1987). Glycolipid binding of purified and recombinant *Escherichia coli* produced verotoxin in vitro.. J Biol Chem.

[33] Waddell T, Head S, Petric M, Cohen A, Lingwood CA (1988). Globotriosylceramide is specifically recognized by the *Escherichia coli* verocytotoxin 2.. Biochem Biophys Res Commun.

[34] DeGrandis S, Law H, Brunton J, Gyles C, Lingwood CA (1989). Globotetraosylceramide is recognized by the pig edema disease toxin.. J Biol Chem.

[35] Samuel JE, Perera LP, Ward S, O'Brien AD, Ginsburg V (1990). Comparison of the glycolipid receptor specificities of Shiga-like toxin type II and Shiga-like toxin type II variants.. Infect Immun.

[36] Bäckhed F, Ahlsén B, Roche N, Ångström J, von Euler A (2002). Identification of target tissue glycosphingolipid receptors for uropathogenic, F1C-fimbriated *Escherichia coli*, and its role in mucosal inflammation.. J Biol Chem.

[37] Jansson L, Tobias J, Jarefjäll C, Lebens M, Svennerholm A-M (2009). The major subunit (CfaB) of colonization factor antigen I from enterotoxigenic *Escherichia coli* is a glycosphingolipid binding protein.. PLoS ONE.

[38] Krivan HC, Olson LD, Barile MF, Ginsburg V, Roberts DD (1989). Adhesion of *Mycoplasma pneumoniae* to sulfated glycolipids and inhibition by dextran sulfate.. J Biol Chem.

[39] Brennan MJ, Hannah JH, Leininger E (1991). Adhesion of *Bordetella pertussis* to sulfatides and to the GalNAcß3Gal sequence found in glycosphingolipids.. J Biol Chem.

[40] Khan AS, Johnston NC, Goldfine H, Schifferli DM (1996). Porcine 987P glycolipid receptors on the intestinal brush borders and their cognate bacterial ligands.. Infect Immun.

[41] Saitoh T, Natomi H, Zhao W, Okuzumi K, Sugano K (1991). Identification of glycolipid receptors for *Helicobacter pylori* by TLC-immunostaining.. FEBS Lett.

[42] Kamisago S, Iwamori M, Tai T, Mitamura K, Yazaki Y (1996). Role of sulphatides in adhesion of *Helicobacter pylori* to gastric cancer cells.. Infect Immun.

[43] Rousset E, Harel J, Dubreuil JD (1998). Sulfatide from the pig jejunum brush border epithelial cell surface is involved in binding of *Escherichia coli* enterotoxin b.. Infect Immun.

[44] Karlsson K-A (1989). Animal glycosphingolipids as membrane attachment sites for bacteria.. Annu Rev Biochem.

[45] Jin LZ, Zhao X (2000). Intestinal receptors for adhesive fimbriae of enterotoxigenic *Escherichia coli* (ETEC) K88 in swine–a review.. Appl Microbiol Biotechnol.

[46] Verdonck F, Cox E, Schepers E, Imberechts H, Joensuu J (2004). Conserved regions in the sequence of the F4 (K88) fimbrial adhesin FaeG suggest a donor strand mechanism in F4 assembly.. Vet Microbiol.

[47] Blattner FR, Plunkett G, Bloch CA, Perna NT, Burland V (1997). The complete genome sequence of *Escherichia coli* K-12.. Science.

[48] Van den Broeck W, Cox E, Goddeeris BM (1999). Receptor-specific binding of purified F4 to isolated villi.. Vet Microbiol.

[49] Karlsson K-A (1987). Preparation of total non-acid glycolipids for overlay analysis of receptors for bacteria and viruses and for other studies.. Meth Enzymol.

[50] Samuelsson BE, Pimlott W, Karlsson K-A (1990). Mass spectrometry of mixtures of intact glycosphingolipids.. Methods Enzymol.

[51] Waldi D, Stahl E (1962). Sprühreagentien für die dünnschicht-chromatographie.. Dünnschicht-Chromatographie.

[52] Karlsson K-A, Lanne B, Pimlott W, Teneberg S (1991). The resolution into molecular species by desorption of glycolipids from thin-layer chromatograms, using combined thin-layer chromatography and fast-atom-bombardment mass spectrometry.. Carb Res.

[53] Ito M, Yamagata T (1989). Purification and characterization of glycosphingolipid-specific endoglycosidases (endoglycoceramidases) from a mutant strain of Rhodococcus sp. Evidence for three molecular species of endoglycoceramidase with different specificities.. J Biol Chem.

[54] Marion D, Wüthrich K (1983). Application of phase sensitive two-dimensional correlated spectroscopy (COSY) for measurements of ^1^H-^1^H spin-spin coupling constants in proteins.. Biochem Biophys Res Commun.

