# Supplementary figures and images for: Erythrocyte and Porcine Intestinal Glycosphingolipids Recognized by F4 Fimbriae of Enterotoxigenic Escherichia coli
Source: PLoS One. 2011 Sep 16;6(9):e23309. doi: 10.1371/journal.pone.0023309 (PMC3174951; doi:10.1371/journal.pone.0023309)

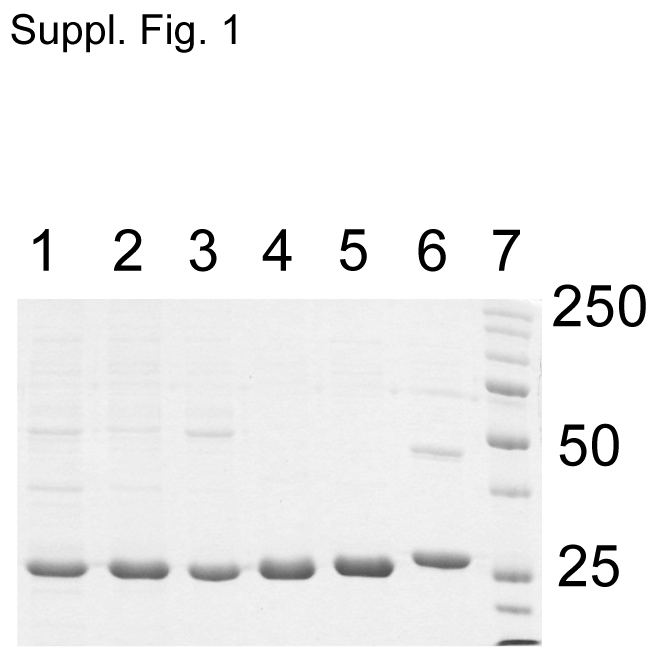

Supplement: Figure S1 — Purified wild type and deletion mutant F4 fimbriae. The protein preparations were separated by SDS-PAGE (12%), and stained by Coomassie Brilliant Blue R-250. The lanes were; Lane 1, F4ab fimbriae with deletion of FaeH, 5 µg; Lane 2, F4ab fimbriae with deletion of FaeI, 5 µg; Lane 3, F4ab fimbriae with deletion of FaeJ, 5 µg; Lane 4, wild type F4ab fimbriae, 5 µg; Lane 5, wild type F4ac fimbriae, 5 µg; Lane 6, wild type F4ad fimbriae, 5 µg; Lane 7, molecular weight marker (kDa). (TIFF) [file pone.0023309.s001.tiff]

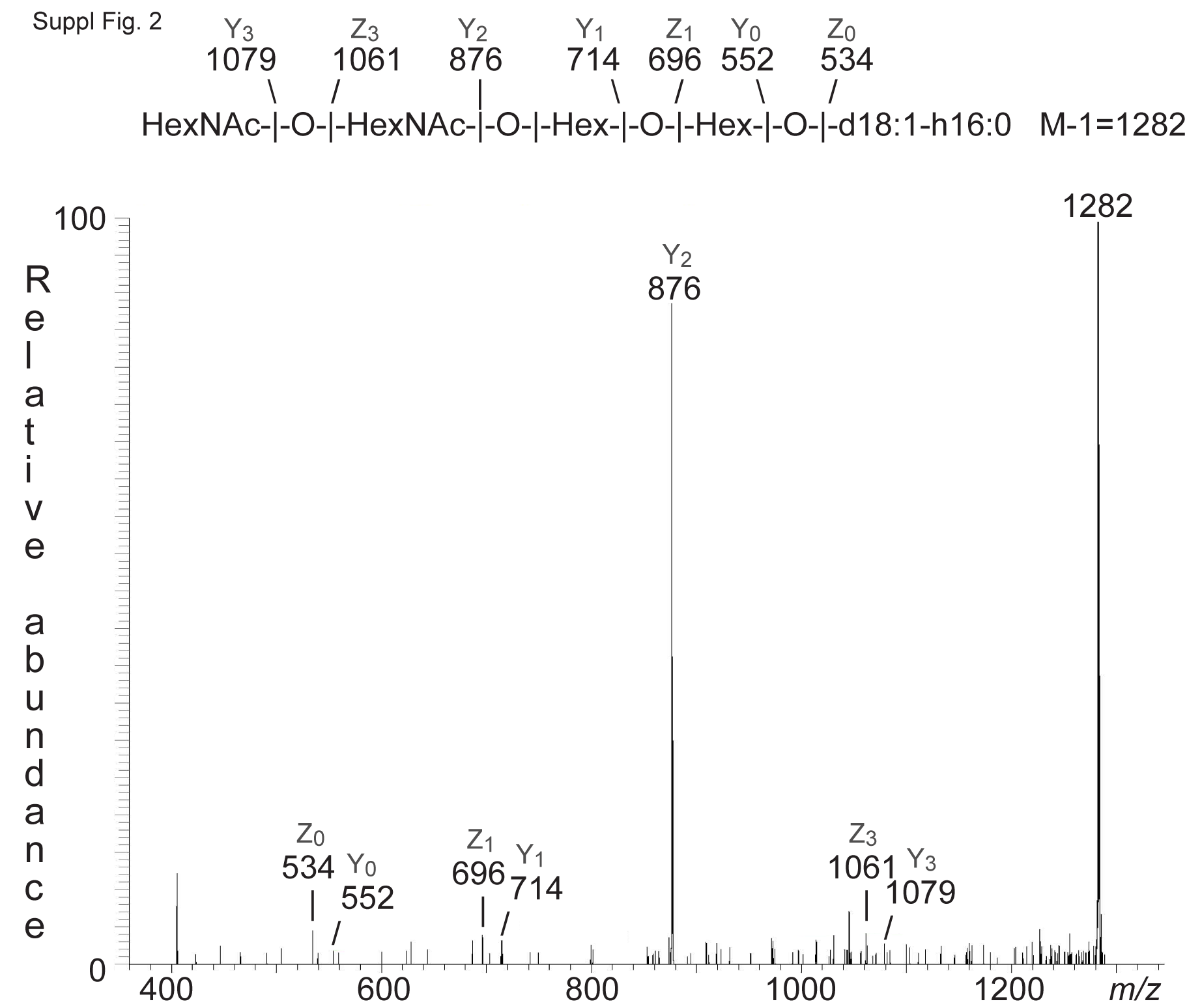

Supplement: Figure S2 — ESI/MS/MS of the native fraction C:Tetra-II from chicken erythrocytes. Above the spectrum is an interpretation formula representing the molecular species with t18:0-h16:0 ceramide. (TIFF) [file pone.0023309.s002.tiff]

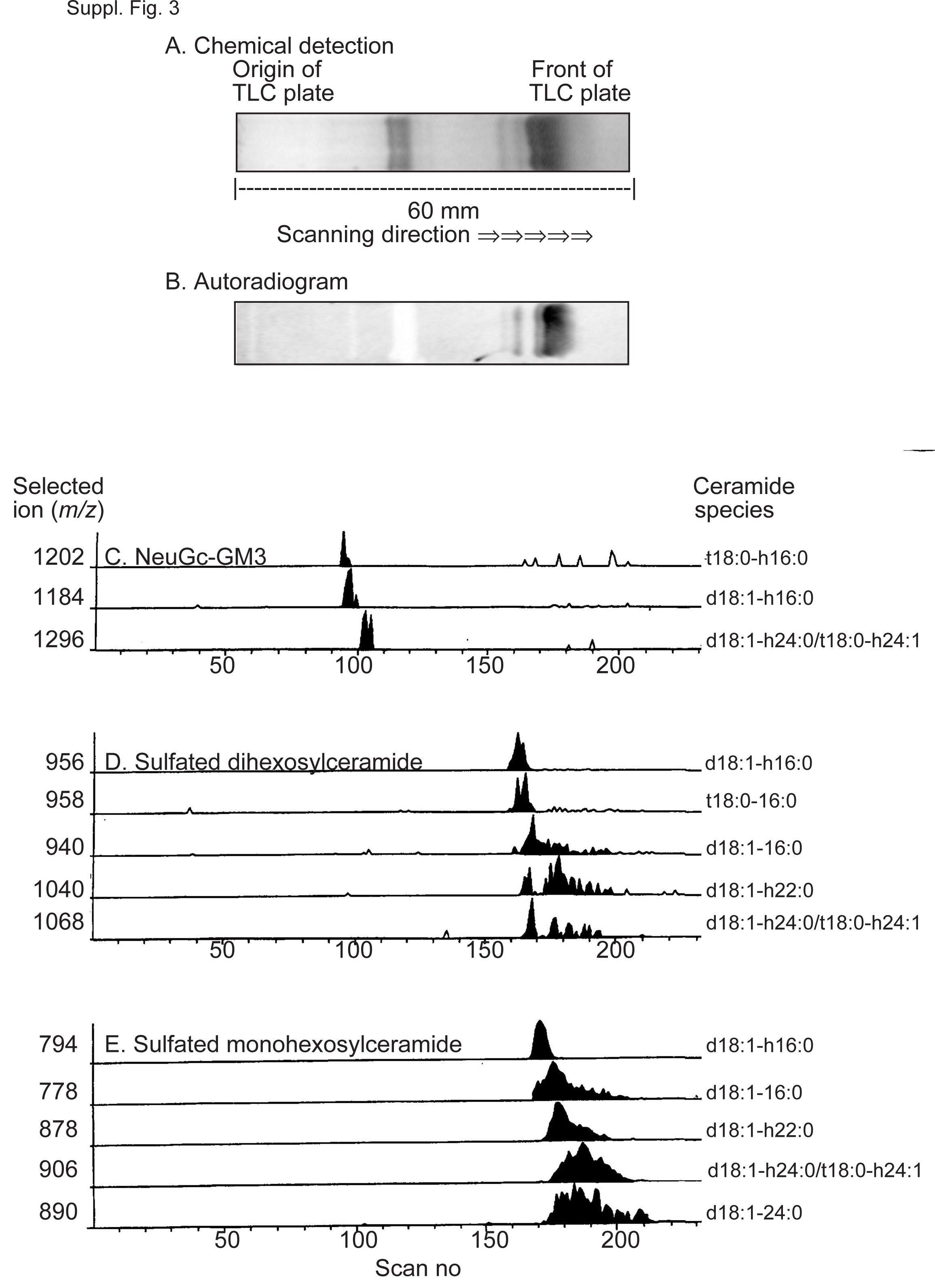

Supplement: Figure S3 — TLC-FAB-MS of the acid glycosphingolipids from newborn piglet small intestinal mucosa. (A) Thin-layer chromatogram stained with anisaldehyde. Acid glycosphingolipids (50 µg) of the epithelial cells of neonatal piglet small intestine were separated on aluminum-backed HPTLC plates using chloroform/methanol/water 60∶35∶8 (by volume) as solvent system. (B) Autoradiogram obtained by binding of 125I-labeled F4ab fimbriae to the acid glycosphingolipids of newborn piglet small intestine. (C–E) Reconstructed curves of selected ions of NeuGc-GM3 (C), sulfated dihexosylceramide (D) and sulfated monohexosylceramide (E), representing the successive detection of different ceramide species. TLC-FAB-MS was performed as described [52], using a ZAB-2F/HF mass spectrometer (VG Analytical, Manchester, UK). Four scans per mm of the thin-layer plate were recorded. The scanning of the thin-layer plate started at the bottom (to the left in the figure) and was run approximately 60 mm upwards, giving in total about 250 scans. After scanning approximately 40 mm of the thin-layer chromatogram, i.e. at the level of the more slow-migrating F4ab-binding glycosphingolipid, peaks appeared that corresponded to molecular ions of sulfated dihexosylceramide. In scans 157–164, the peak at m/z 956, corresponding to the species with d18:1-h16:0, was dominating, and in scan 165 the species with t18:0-16:0 (m/z 958) dominated. The following scans had peaks corresponding to d18:1-16:0 (m/z 940), d18:1-h22:0 (m/z 1040), and d18:1-h24:0 or t18:0-24:1 (m/z 1068) ceramides. At the level of the more fast-migrating F4ab-binding compound (scans 168–211) molecular weight ions of sulfated monohexosylceramide were obtained. Here, ions corresponding to sulfated monohexosylceramide with d18:1-h16:0 (m/z 794), d18:1-16:0 (m/z 778), d18:1-h22:0 (m/z 878), d18:1-h24:0 or t18:0-24:1 (m/z 906), and d18:1-24:0 (m/z 890) were found. Thus, the more slow-migrating F4ab-binding glycosphingolipid was tentatively identified as sulf [file pone.0023309.s003.tiff]

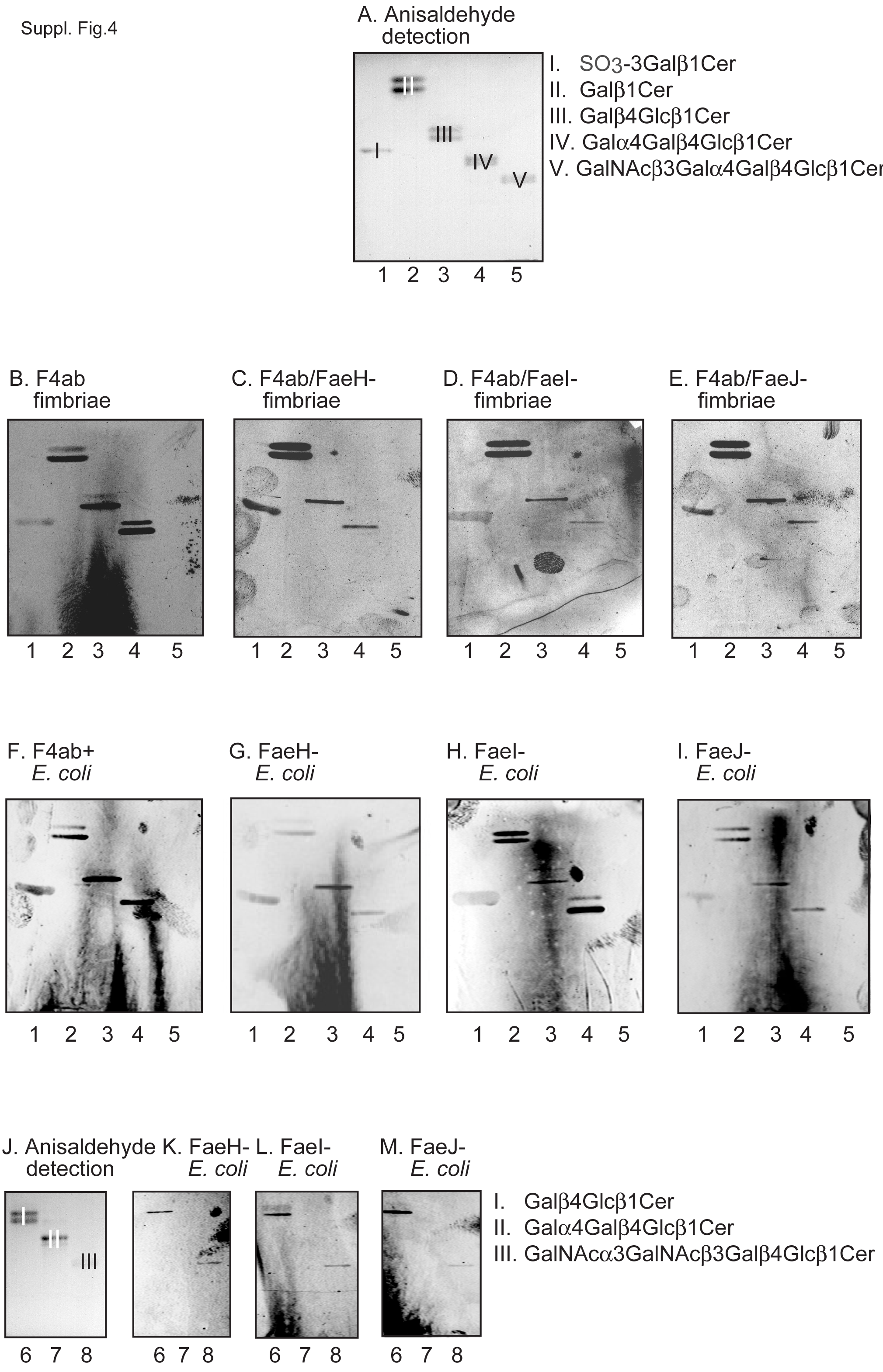

Supplement: Figure S4 — Binding of deletion mutant F4ab fimbriae to glycosphingolipids. Thin-layer chromatograms after chemical detection by anisaldehyde (A and J), and autoradiograms obtained by binding of 35S-labeled native F4ab fimbriae (B), F4ab fimbriae with deletions of FaeH (C), FaeI (D) and FaeJ (E), and 35S-labeled E. coli expressing native F4ab fimbriae (F), and F4ab fimbriae with deletions of FaeH (G and K), FaeI (H and L) and FaeJ (I and M). The glycosphingolipids were separated on aluminum-backed silica gel plates, using chloroform/methanol/water (60∶35∶8, by volume) as solvent system, and the binding assays were performed as described under “Materials and Methods”. Autoradiography was for 12 h. The lanes on A-I were: Lane 1, Sulfatide (SO3-3Galß1Cer) with t18:0-h24:0 ceramide, 4 µg; Lane 2, Galactosylceramide (Galß1Cer) with d18:1-h18:0-h24:0 ceramide, 4 µg; Lane 3, Lactosylceramide (Galß4Glcß1Cer) with t18:0-h16:0-h24:0 ceramide, 4 µg; Lane 4, globotriaosylceramide (Galα4Galß4Glcß1Cer) with t18:0-22:0-24:0 ceramide, 4 µg; Lane 5, globotetraosylceramide (GalNAcß3Galα4Galß4Glcß1Cer) with t18:0-h16:0-h24:0 ceramide, 4 µg. The lanes on J-M were: Lane 6, Lactosylceramide (Galß4Glcß1Cer) with t18:0-h16:0-h24:0 ceramide, 4 µg; Lane 7, globotriaosylceramide (Galα4Galß4Glcß1Cer) with d18:1-16:0 and d18:1-24:0 ceramide, 4 µg; Lane 8, GalNAcα3GalNAcß3Galß4Glcß1Cer of chicken erythrocytes, 4 µg. The glycosphingolipids visualized with anisaldehyde in (A) and (J) are marked with Roman numbers, and the corresponding glycosphingolipid structures are are given to the right of the chromatograms. (TIFF) [file pone.0023309.s004.tiff]
