# Supplementary material for: Erythrocyte and Porcine Intestinal Glycosphingolipids Recognized by F4 Fimbriae of Enterotoxigenic Escherichia coli
Source: PLoS One. 2011 Sep 16;6(9):e23309. doi: 10.1371/journal.pone.0023309 (PMC3174951; doi:10.1371/journal.pone.0023309)
Supplement: Table S1 — Summary of results from binding of F4 fimbriae and F4-fimbriated Escherichia coli to glycosphingolipids on thin-layer chromatograms. (DOC) [file pone.0023309.s005.doc]

**Table S1.** Binding of F4 fimbriae and F4-fimbriated *Escherichia coli* to glycosphingolipids on thin-layer chromatograms

| **No.** | **Trivial name** | **Structure** | **F4aba** | **F4aca** | **F4ada** | **Source** |
| --- | --- | --- | --- | --- | --- | --- |
| *Simple compounds* |  |  |  |  |  |  |
| 1. | Galactosylceramide (t18:0-h24:0) | Galß1Cer | +++b | +++ | - | Porcine intestine |
| 2. | Glucosylceramide (d18:1-h24:0) | Glcß1Cer | - | - | - | Porcine kidney |
| 3. | Sulfatide (d18:1-h16:0) | SO_3_-3Galß1Cer | +++ | + | - | Human intestine |
| 4. | Sulfatide (d18:1-24:1) | SO_3_-3Galß1Cer | +++ | + | - | Human intestine |
| 5. | Sulfatide (t18:0-h24:0) | SO_3_-3Galß1Cer | +++ | + | - | Human intestine |
| 6. | Lactosylceramide (d18:1-16:0-24:1) | Galß4Glcß1Cer | - | - | - | Human neutrophils |
| 7. | Lactosylceramide (t18:0-h16:0-h24:0) | Galß4Glcß1Cer | +++ | +++ | +++ | Dog intestine |
| 8. | Sulf-lactosylceramide (t18:0-h16:0) | SO_3_-3Galß4Glcß1Cer | +++ | - | - | Human kidney |
| 9. | Galabiaosylceramide (d18:1-16:0-18:0) | Galα4Galß1Cer | +++ | - | - | Synthetic |
| 10. | Lactotriaosylceramide (d18:1:16:0/24:1) | GlcNAcß3Galß4Glcß1Cer | - | - | - | Human neutrophils |
| 11. | (d18:1-h16:0) | GalNAcα3GalNAcß3Galß4Glcß1Cer | +++ | +++ | +++ | Chicken erythrocytes |
| 12. | (d18:1-h16:0) | GalNAcα3GalNAcß3Galα4Galß1Cer | NDc | NDc | NDc | Chicken erythrocytes |
| *Ganglioseries* |  |  |  |  |  |  |
| 13. | Gangliotriaosylceramide (d18:1-16:0/24:0) | GalNAcß4Galß4Glcß1Cer | - | - | +++ | Guinea pig erythrocytes |
| 14. | Gangliotetraosylceramide (t18:0-h16:0/h24:0) | Galß3GalNAcß4Galß4Glcß1Cer | - | - | +++ | Mouse intestine |
| 15. | Sulf-gangliotetraosylceramide (t18:0-h16:0/h24:0) | SO_3_-3Galß3GalNAcß4Galß4Glcß1Cer | - | - | - | Mouse intestine |
| *Neolactoseries* |  |  |  |  |  |  |
| 16. | Neolactotetraosylceramide (d18:1-16:0/24:1) | Galß4GlcNAcß3Galß4Glcß1Cer | - | - | + | Human neutrophils |
| 17. | H type 2 pentaglycosylceramide (d18:1-16:0/24:0) | Fucα2Galß4GlcNAcß3Galß4Glcß1Cer | - | - | - | Human erythrocytes |
| 18. | B5 pentaglycosylceramide (d18:1-16:0/24:0) | Galα3Galß4GlcNAcß3Galß4Glcß1Cer | - | - | - | Rabbit erythrocytes |
| 19. | P1 pentaglycosylceramide (d18:1-16:0/24:0) | Galα4Galß4GlcNAcß3Galß4Glcß1Cer | - | - | - | Human erythrocytes |
| 20. | Lex pentaglycosylceramide (t18:0-h16:0-h24:0) | Galß4(Fucα3)GlcNAcß3Galß4Glcß1Cer | - | - | - | Dog intestine |
| 21. |  | GalNAcα3GalNAcß3Galß4GlcNAcß3Galß4Glcß1Cer | +++ | +++ | +++ | Chicken erythrocytes |
| 22. | B type 2 hexaglycosylceramide (d18:1-16:0/24:0) | Galα3(Fucα2)Galß4GlcNAcß3Galß4Glcß1Cer | - | - | - | Human erythrocytes |
| 23. | A type 2 hexaglycosylceramide (d18:1-16:0/24:0) | GalNAcα3(Fucα2)Galß4GlcNAcß3Galß4Glcß1Cer | - | - | - | Human erythrocytes |
| *Lactoseries* |  |  |  |  |  |  |
| 24. | Lactotetraosylceramide (d18:1/t18:0-h16:0-h24:0) | Galß3GlcNAcß3Galß4Glcß1Cer | - | - | - | Human meconium |
| 25. | Lea pentaglycosylceramide (t18:0-h16:0-h24:0) | Galß3(Fucα4)GlcNAcß3Galß4Glcß1Cer | - | - | - | Human meconium |
| 26. | Leb hexaosylceramide (t18:0-h16:0-h24:0) | Fucα2Galß3(Fucα4)GlcNAcß3Galß4Glcß1Cer | - | - | - | Human meconium |
| 27. | H type 1 pentaglycosylceramide (t18:0-h16:0-h24:0) | Fucα2Galß3GlcNAcß3Galß4Glcß1Cer | - | - | - | Porcine intestine |
| 28. | B type 1 hexaglycosylceramide (t18:0-h16:0-h24:0) | Galα3(Fucα2)Galß3GlcNAcß3Galß4Glcß1Cer | - | - | - | Human intestine |
| 29. | A type 1 hexaglycosylceramide (t18:0-h16:0-h24:0) | GalNAcα3(Fucα2)Galß3GlcNAcß3Galß4Glcß1Cer | - | - | - | Human intestine |
| *Globoseries* |  |  |  |  |  |  |
| 30. | Isoglobotriaosylceramide (t18:0-h22:0/h24:0) | Galα3Galß4Glcß1Cer | - | - | + | Cat intestine |
| 31. | Globotriaosylceramide (d18:1-16:0-24:0) | Galα4Galß4Glcß1Cer | - | - | - | Human erythrocytes |
| 32. | Globotriaosylceramide (t18:0-h22:0-h24:0) | Galα4Galß4Glcß1Cer | +++ | - | - | Rat intestine |
| 33. | Globotriaosylceramide (t18:0-h24:0) | Galα4Galß4Glcß1Cer | +++ | - | - | Pig intestine |
| 34. | Globoside (d18:1-16:0/24:0) | GalNAcß3Galα4Galß4Glcß1Cer | - | - | - | Human erythrocytes |
| 35. | Globoside (t18:0-h16:0-h24:0) | GalNAcß3Galα4Galß4Glcß1Cer | - | - | - | Human meconium |
| 36. | Forssman pentaglycosylceramide (d18:1-16:0/24:0) | GalNAcα3GalNAcß3Galα4Galß4Glcß1Cer | - | - | - | Dog intestine |
| 37. | para-Forssman pentaglycosylceramide (d18:1-16:0/24:0) | GalNAcß3GalNAcß3Galα4Galß4Glcß1Cer | - | - | - | Human erythrocytes |
| *Gangliosides* |  |  |  |  |  |  |
| 38. | NeuGc-GM3 (d18:1-h16:0-h24:0) | NeuGcα3Galß4Glcß1Cer | - | - | - | Piglet intestine |
| 39. | NeuAc-GM3 (d18:1-18:0/d20:1-18:0) | NeuAcα3Galß4Glcß1Cer | - | - | - | Human brain |
| 40. | NeuGc-GM1 (d18:1-h16:0-h24:0) | Galß3GalNAcß4(NeuGcα3)Galß4Glcß1Cer | - | - | - | Calf intestine |
| 41. | NeuAc-GM1(d18:1-18:0/d20:1-18:0) | Galß3GalNAcß4(NeuAcα3)Galß4Glcß1Cer | - | - | - | Human brain |
| 42. | NeuAc-GD1a(d18:1-18:0/d20:1-18:0) | NeuAcα3Galß3GalNAcß4(NeuAcα3)Galß4Glcß1Cer | - | - | - | Human brain |
| 43. | NeuAcα3-neolactotetraosylceramide (d18:1-16:0/24:0) | NeuAcα3Galß4GlcNAcß3Galß4Glcß1Cer | - | - | - | Human erythrocytes |

aF4ab denotes bindings obtained with both F4ab fimbriae and F4ab-fimbriated *E. coli*, F4ac bindings obtained with both F4ac fimbriae and F4ac-fimbriated *E. coli*, and F4ad bindings obtained

with both F4ad fimbriae and F4ad-fimbriated *E. coli*.

bBinding is defined as follows: +++ denotes an intense and highly reproducible staining when 4 µg of the glycosphingolipid was applied on the thin-layer chromatogram, + denotes an occasional

staining,while - denotes no binding even at 4 µg.

cND, not determined.
